# Pyrolysis and Combustion Chemistry of Pyrrole, a Reference Component for Bio-oil Surrogates: Jet-Stirred Reactor Experiments and Kinetic Modeling

**DOI:** 10.1021/acs.energyfuels.0c03874

**Published:** 2021-03-02

**Authors:** Matteo Pelucchi, Suphaporn Arunthanayothin, Yu Song, Olivier Herbinet, Alessandro Stagni, Hans-Heinrich Carstensen, Tiziano Faravelli, Frédérique Battin-Leclerc

**Affiliations:** † CRECK Modeling Lab, Department of Chemistry Materials and Chemical Engineering, Politecnico di Milano, 20133 Milano, Italy; ‡ Laboratoire Réactions et Génie des Procédés, CNRS, Université de Lorraine, ENSIC, 54001 Nancy Cedex, France; § University Orléans, INSA-CVL, PRISME, EA 4229, 45072 Orléans, France; ∥ Fundación Agencia Aragonesa para la Investigación y Desarrollo (ARAID), 50018 Zaragoza, Spain; ⊥ Department of Chemical and Environmental Engineering, Engineering and Architecture School, University of Saragoza, 50018 Zaragoza, Spain

## Abstract

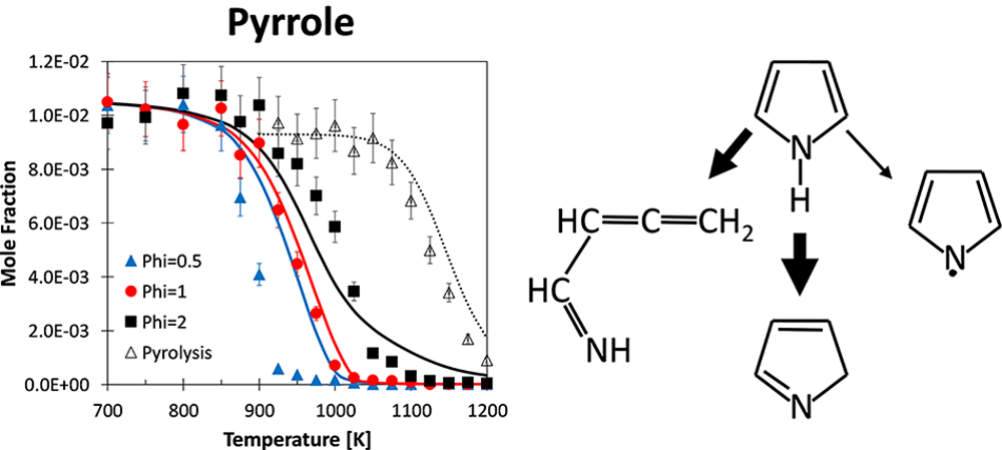

Fast-pyrolysis bio-oils (FPBOs) obtained from lignocellulosic biomass are gaining attention as sustainable fuels for various applications, including the transport sector and power production. A significant fraction of bio-oils is constituted by nitrogen-containing compounds (N fuels) that should be considered when developing surrogate models for FPBOs. Moreover, the content of N fuels in FPBOs is expected to strongly contribute to the production of nitrogen oxides (NO_*x*_) directly from fuel-bound nitrogen (fuel NO_*x*_), in addition to the thermal NO_*x*_ formation pathways typical of high-temperature combustion conditions. This work investigates the pyrolysis and combustion chemistry of pyrrole (C_4_H_5_N), a candidate reference fuel component for FPBO surrogate models. Speciation measurements in an atmospheric pressure jet-stirred reactor have been performed for both pyrolysis and oxidation conditions. Pyrolysis experiments have been performed for 1% pyrrole/helium mixtures over the temperature range *T* = 925–1200 K. Oxidation experiments were carried out for 1% pyrrole/oxygen/helium mixtures at three equivalence ratios (φ = 0.5, 1.0, and 2.0) over the temperature range *T* = 700–1200 K. These new data significantly extend the number of experimental targets for kinetic model validation available at present for pyrrole combustion. After a thorough revision of previous theoretical and kinetic modeling studies, a preliminary kinetic model is developed and validated by means of comparison to new experimental data and those previously reported in the literature. The rate of production and sensitivity analyses highlight important pathways deserving further investigations for a better understanding of pyrrole and, more in general, N fuel combustion chemistry. A critical discussion on experimental challenges to be faced when dealing with pyrrole is also reported, encouraging further experimental investigation with advanced diagnostics.

## 1 Introduction

Concerns about climate change and energy security are pushing industries and academia to seek alternatives to fossil fuels, pursuing a more sustainable energy scenario. The European Green Deal^1^ recently set a roadmap of the key policies and measures needed to meet the United Nations 2030 agenda^2^ in terms of sustainable development goals, aiming at zero net emissions of greenhouse gases by 2050.

Within the different alternatives (e.g., electrification of the transport sector, hydrogen energy, electrofuels, nuclear energy, and hydroelectricity), fast pyrolysis is an effective and promising process to obtain high yields of bio-oils from lignocellulosic biomass. Downstream upgrading of fast-pyrolysis bio-oils (FPBOs) provides valuable fuels for transport and chemicals for industry.^3^ A great advantage of such a conversion process is that bio-oils have ∼5–20 times higher volumetric energy density compared to biomass feedstocks, facilitating transport and distribution to a centralized location for use as feedstock for further downstream processing (e.g., gasification/Fischer–Tropsch synthesis, catalytic hydrotreatment, catalytic cracking, and hydrodeoxygenation^4,5^), therefore driving the development of a sustainable market for lignocellulosic biomass. Bio-oils are very different from fossil fuels in terms of both physical and chemical properties, posing some technical challenge for their effective implementation in existing distribution infrastructures and combustion systems typically used for power or heat generation and in the transport sector [e.g., internal combustion (IC) engines and jet engines]. Indeed, bio-oils are typically non-flammable or non-distillable acidic fuels (pH ∼ 2–3) with a high water fraction (15–30 wt %), high oxygen content (∼30 wt % on a dry basis), and significant inorganic fraction (metal, ash, char, and solid particles). Such properties negatively affect both the viability of downstream upgrading processes and the direct use of FPBO in combustion processes as a result of generally low heating values, low propensity to ignition, low thermal stability, material incompatibility, corrosion, immiscibility with other hydrocarbon streams, possible fuel pump and nozzle clogging (e.g., in sprays), etc.^6^ However, as recently reviewed by Letho et al., accounting for FPBO upgrading and relatively minor technical and operational adjustments (e.g., material selection, air and fuel preheating, co-feeding with support fuel, or feeding to a pilot flame) already allowed for successful testing of FPBOs in large-scale burners, gas turbines, and compression ignition engines for heat and/or power generation.^6^

The key to the solution of the technical challenges related to FBPO use in combustors and IC engines is the knowledge of high- and low-temperature combustion chemistry of FPBOs and their upgraded streams. Indeed, chemical kinetics plays the major role in understanding and optimizing combustion processes, for improved efficiency, improved fuel economy, and reduced pollution.^7^ Because it is typical for complex liquid fuels, such as FPBOs, a fuel model requires first the definition of a limited number of reference species accounted for in the surrogate fuel model. For each of these species, a kinetic subset together with thermodynamic and transport properties is then required to describe pyrolysis and high- and low-temperature combustion phenomena, such as ignition, flame propagation, and pollutant formation. FPBOs contain hundreds of organic compounds, such as phenolic components, aldehydes, alcohols, acids, esters, anhydrosugars, furans, and nitrogen-containing compounds, as well as large anhydro-oligosaccharides and lignin-derived oligomers.^8^ From a pure combustion chemistry perspective, each of these chemical families should be taken into account when formulating a suitable fuel surrogate because each of them carry a specific reactivity strongly related to specific functional groups and the molecular structure^9^ that can be determining in properly predicting the macroscopic target of interest for large-scale applications.

As reported in recent studies, the CRECK kinetic framework was extended to describe a large number of chemical families found in FPBOs. Kinetic subsets for phenol and phenolic compounds (e.g., anisole, catechol, guaiacol, and vanillin) have been recently presented by Pratali Maffei et al.^10^ and Pelucchi et al.^11^ Aldehyde chemistry has been recently discussed in refs (12 and 13), with the latter study specifically focusing on benzaldehyde, the simplest aromatic aldehyde. Alcohol chemistry has been systematically investigated in refs (14 and 15). Kinetic subsets for acetic, butanoic, and pentanoic acids have also been developed and validated.^16,17^ Previous studies by Grana et al.,^18,19^ Saggese et al.,^20^ and Rodriguez et al.^21^ addressed the chemistry of methyl esters and fatty acid methyl esters (FAMEs), both of interest as surrogate components for FPBOs and biodiesel fuels (e.g., FAMEs).

This work presents a further extension of the fuel palette of the CRECK kinetic framework^22^ to describe the pyrolysis and combustion kinetics of a nitrogen-containing fuel (N fuel): pyrrole (C_4_H_5_N). Beside the mere necessity of representing the N fuel fraction of FPBOs, understanding pyrrole combustion kinetics is also of relevance for a better assessment of fuel NO_*x*_, namely, the fraction of nitrogen oxides (NO_*x*_) formed during the oxidation of nitrogen contained in a fuel molecule in a combustion environment.^23,24^ Fuel NO_*x*_ are of interest in not only FPBO combustion but also biomass and coal combustion. Indeed, biomass and coal first undergo devolatilization processes,^25,26^ during which a part of fuel-bound nitrogen is devolatilized and pyrolyzed into NO_*x*_ precursors, such as hydrogen cyanide (HCN), ammonia (NH_3_), and isocyanic acid (HNCO),^23,24^ which can be further converted into NO, N_2_O, and N_2_ as final products. In this regard, this work extends our recently revised model for thermal and prompt NO_*x*_ formation as well as high-temperature NO_*x*_ reburning phenomena.^27^

## 2 Previous Experimental, Theoretical, and Kinetic Modeling Studies on Pyrrole Pyrolysis and Oxidation

A very limited number of theoretical, experimental, and kinetic modeling studies has been reported for pyrrole in previous studies. Structures and names of the chemical species related to pyrrole chemistry and used in the following discussion are reported in Table 1 to facilitate the reading.

**Table 1:**
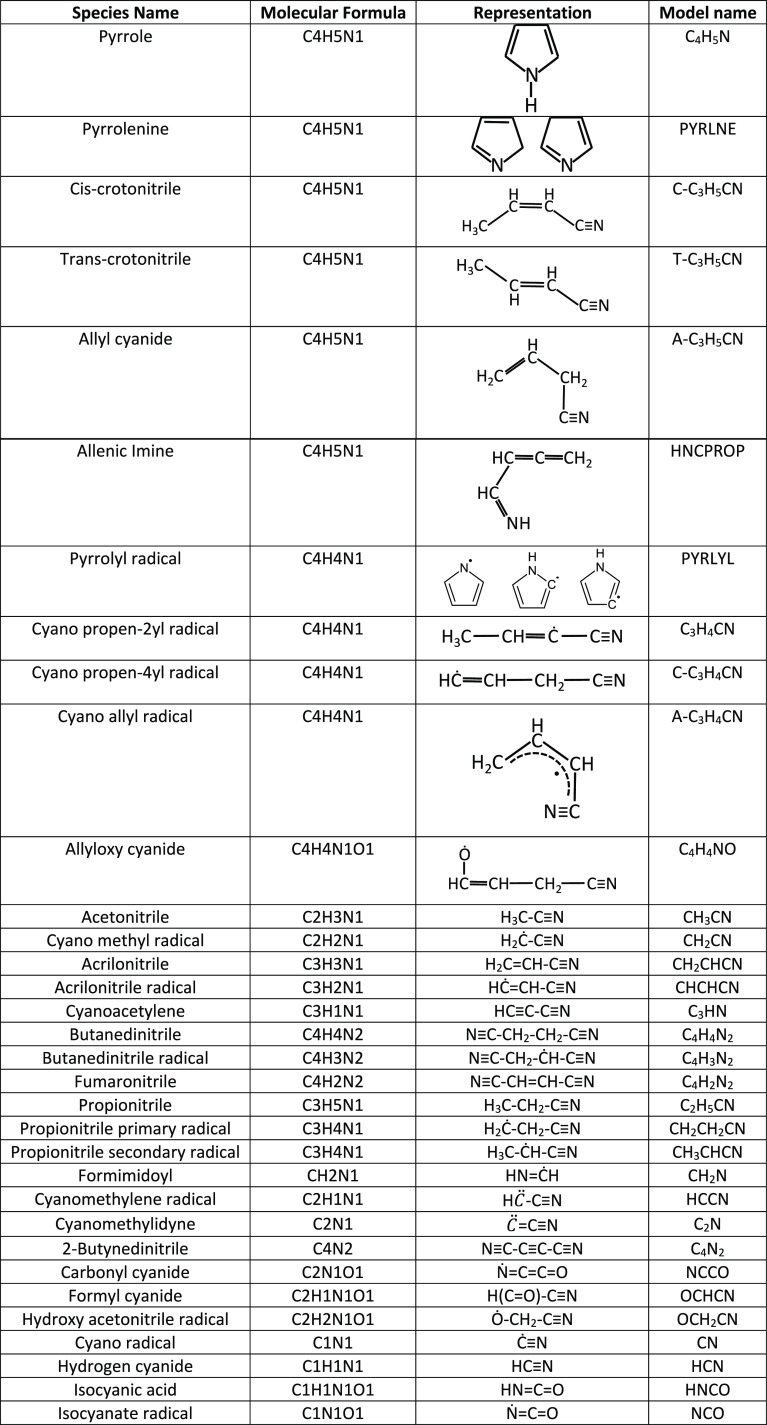
Nomenclature of Relevant Species in Pyrrole Pyrolysis and Oxidation^a^

a Thermodynamic properties are reported in Table S1 of the Supporting Information and compared to literature values where available. Simplified molecular-input line-entry system (SMILES) identifiers are reported in Table S2 of the Supporting Information.

Lifshitz et al.^28^ investigated the thermal decomposition of pyrrole in a single-pulse shock tube at *T* = 1050–1450 K and *p* = 2.6–3.6 atm (i.e., overall density of ∼3 × 10^–5^ mol/cm^3^). Pyrrole was found to mainly isomerize to *cis*-crotonitrile (*c*C_3_H_5_CN) and allyl cyanide (aC_3_H_5_CN) or decompose to propyne (C_3_H_4_-p) and hydrogen cyanide (HCN), in relative ratios of 58, 25, and 17%. Secondary reactivity of these major products yields other species measured in significant quantities, such as acetonitrile (CH_3_CN), acetylene (C_2_H_2_), allene (C_3_H_4_-a), methane (CH_4_), and ethylene (C_2_H_4_). Mackie et al.^29^ studied the kinetics of the pyrolysis of highly diluted pyrrole/argon mixtures (0.07 and 0.5 mol % pyrrole) in a single–pulse shock tube at *T* = 1200–1700 K and *p* = 7.5–13 atm. A kinetic model composed of 75 elementary steps was presented and found to largely reproduce the experimental observations. The authors suggested that the thermal decomposition of pyrrole is initiated by the reversible isomerization to pyrrolenine (2*H*-pyrrole, PYRLNE), occurring through a 1–2 hydrogen shift. The C–N bond fission in pyrrolenine then leads to ring opening, forming a biradical that is rapidly transformed in *cis*-crotonitrile or allyl cyanide. H-abstraction reactions by Ḣ and ĊH_3_ also contribute to pyrrole consumption, forming the resonance-stabilized pyrrolyl radical and secondary products, such as hydrogen (H_2_) and methane. Dubnikova and Lifshitz^30^ theoretically investigated the isomerization pathways of pyrrole using the density functional theory (B3LYP/cc-pvDZ). The theoretical analysis confirmed the previous findings by Mackie et al.,^29^ according to which the decomposition of pyrrole is initiated by the fast isomerization to pyrrolenine through a 1,2-H migration, reaching a state of equilibrium from which ring-opening and isomerization reactions to *cis*-crotonitrile (*c*C_3_H_5_CN) and allyl cyanide (aC3H5CN) occur. Rice–Ramsperger–Kassel–Marcus (RRKM) calculations were performed, and the master equation (ME) solved for the highest energy barrier (*E*_a_) steps, i.e., for the first isomerization (C_4_H_5_N ↔ PYRLNE; *E*_a_ = 43.1 kcal/mol) and for the ring-opening reaction leading to a biradical intermediate (PYRLNE ↔ Ṅ=CH–CH=CH–ĊH_2_; *E*_a_ = 68.0 kcal/mol). This intermediate can further isomerize to allyl cyanide (*E*_a_ = 16.3 kcal/mol) or to a less stable conformer (*E*_a_ = 4.1 kcal/mol) accessible by rotating the N=CH moiety. This conformer can then isomerize to *cis*-crotonitrile (*E*_a_ = 7.7 kcal/mol) and *trans*-crotonitrile (*t*C_3_H_5_CN). Optimized geometries and frequencies for biradical structures were performed using the unrestricted uB3LYP with the same basis set and also optimized with a complete active space multiconfiguration self-consistent field (CASSCF) with CAS(4,4) wave functions for comparison. Each optimized SCF structure was recalculated at a single-point quadratic CI, including single and double substitutions with a triplet contribution to the energy, QCISD(T), using uB3LYP structures as the first guess. An additional theoretical investigation of the pyrolysis mechanisms of pyrrole was presented by Zhai et al.^31^ Equilibrium and transition state structures of the proposed reaction channels were fully optimized by the density functional B3LYP method using the 6-31G(d,p) basis set, and relative energies were evaluated at the QCISD(T)/6-311G(d,p) level of theory or with the unrestricted equivalents for biradical structures [uB3LYP and uQCISD(T)]. Good agreement was obtained in terms of optimized structures, but the channels leading to allyl cyanide turned out to be much higher in energy compared to the previous calculations as a result of both the different basis sets and some inconsistencies in the calculation methods adopted by Dubnikova and Lifshitz,^30^ as highlighted by Zhai et al.^31^ The latter study proposed an additional closed-shell pathway, not involving the biradical structures, for the formation of *cis*-crotonitrile. Two low-energy closed-shell intermediates are successively formed from pyrrolenine: 3*H*-pyrrole (pyrrolenine ↔ 3*H*-pyrrole; *E*_a_ = 28.2 kcal/mol) through a second 1,2-H migration and *cis*-isocyanocrotonitrile (3*H*-pyrrole ↔ *cis*-isocyanocrotonitrile; *E*_a_ = 68.3 kcal/mol) via a concerted transition state, including a C–C bond cleavage and a 1,2-hydrogen migration. The authors concluded that the new low-energy pathway might compete with that investigated by Dubnikova and Lifshitz at low-pressure conditions (i.e., <1 atm), but at a higher pressure the open-shell channel forming the biradical structures dominates the decomposition chemistry as a result of collisional deactivation of the more stable intermediates. Extensive calculations were carried out in the same study seeking decomposition pathways directly generating HCN, as highlighted by the experimental measurements of Dubnikova and Lifshitz.^30^ However, no such competitive pathway was identified. Martoprawiro et al.^32^ investigated the pyrolysis kinetics of pyrrole, including the thermochemistry of relevant species, by means of CASSCF, CASPT2, and G2(MP2) calculations. In addition to the two major decomposition channels proceeding through pyrrolenine already reported in the previous studies, a third channel involving the fission of a H atom and formation of the resonance-stabilized cyanoallyl radical (aĊ_3_H_4_CN, ĊH_2_–CH=CH–C≡N) was identified. An additional channel forming an allenic imine (HN=CH–CH=C=CH_2_, HCNPROP) contributes to the formation of HCN and propyne. The lowest energy pathway involves the isomerization of pyrrole to a cyclic carbene through a 4,5-H migration, and successive ring opening produces the allenic imine intermediate.^33^ On the basis of the calculated rate constants and the previous studies briefly reviewed above, Martoprawiro et al.^32^ proposed a kinetic model validated by comparison to the experimental pyrolysis data by Mackie et al.^29^ Hong et al.^34^ performed experimental measurements for the pyrolysis of pyrrole (6.46 mol % in argon) with the tunable synchrotron vacuum ultraviolet (VUV) photoionization and molecular-beam mass spectrometry (MBMS) technique in a pyrolysis chamber located in a high-temperature furnace. The measurements were carried out at *p* = 0.002 atm, over the temperature range *T* = 1260–1710 K. Formation pathways of the major products (HCN, C_2_H_2_, CH_3_CN, and C_3_H_4_-p) and radical intermediates (aĊ_3_H_4_CN and ĊH_2_CN) were investigated using the composite G3B3 method, highlighting the lowest energy formation pathways. The HCN formation channel proceeding through cyclic carbene discussed above was proposed to be most favored, in accordance with the previous theoretical work by Martoprawiro et al.^32^

Assuming complete oxidation of molecular nitrogen to nitrogen dioxide (NO_2_), the combustion of pyrrole can be defined by the following reaction equation:^35^
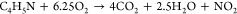
Lumbreras et al.^36^ presented the first experimental and kinetic modeling study on pyrrole oxidation. Experimental measurements were carried out in an isothermal quartz flow reactor at atmospheric pressure in the temperature range *T* = 700–1500 K for diluted mixtures of pyrrole (0.01 mol %), oxygen (∼0.05–1.37 mol %), and water (∼1.0–1.2 mol %). The effects of the temperature, equivalence ratio (φ = 0.04, 0.90, and 1.18, where φ is defined from the above reaction equation), and NO addition (∼0.3 mol %) on CO, CO_2_, HCN, and NO concentrations were evaluated experimentally and modeled by means of a preliminary kinetic model. Rate constants were adopted from previous studies for pyrolysis pathways^28−33^ and based on analogy with phenol/phenoxy chemistry. Ignition delay times of diluted mixtures of pyrrole (0.5 and 1.0 mol %) and oxygen in argon were measured in a low-pressure shock tube by MacNamara and Simmie.^35^ Ignition measurements were performed in the temperature range *T* = 1102–1805 K, pressures *p* = 220–520 kPa, and equivalence ratios φ = 0.5, 1.0, and 2.0. Koger and Bockhorn^37^ investigated the formation of HCN from the oxidation of pyrrole under incinerator conditions (*T* = 1180 and 1220 K, *p* = 1 atm, and φ = 0.81 and 1.04) in a turbulent flow reactor. Tian et al.^38^ investigated the oxidation of pyrrole, oxygen, and argon mixtures (φ = 0.55 and 1.84) in premixed laminar flames at *p* = 0.032 atm using tunable synchrotron photoionization and MBMS techniques. Results highlighted that N_2_, NO, and NO_2_ are the major nitrogen-containing products, while hydrogen cyanide, isocyanic acid (HNCO), and 2-propenenitrile (CH_2_CHCN) are the most important nitrogen-containing intermediates. The formation of fuel NO from pyrrole oxidation was studied by Yamamoto et al.^39^ in a quartz flow reactor in the temperature range *T* = 800–1400 K at atmospheric pressure. The inlet concentration of pyrrole (0.02 mol %) was kept constant while varying the O_2_ content (0.64 and 2.00 mol %) and water content (3 and 8 mol %). The impact of the residence time was also assessed for the mixture containing 2.00 mol % oxygen and 8 mol % water. A kinetic model composed of 89 chemical species and 505 reactions was proposed on the basis of the previous pyrolysis model by Mackie et al.^29^ and the oxidation pathways proposed by Lumbreras et al.^36^

The present work reports new experimental measurements for pyrrole pyrolysis and oxidation in an atmospheric pressure jet-stirred reactor (JSR), significantly extending the scarce validation targets presented in the literature thus far. Pyrolysis experiments have been performed in the temperature range *T* = 925–1200 K for mixtures of ∼1.0 mol % pyrrole in helium. Oxidation experiments have been performed in the temperature range *T* = 700–1200 K for 1.0% pyrrole/O_2_/He mixtures at variable equivalence ratios (φ = 0.5, 1.0, and 2.0).

In addition, a preliminary model is presented on the basis of previous theoretical, experimental, and kinetic modeling efforts. Thermodynamic data not reported in previous studies or not available in thermodynamic property databases have also been calculated in this work. To the best of our knowledge, this model represents the first comprehensively validated model reported in the literature thus far and, despite evident shortcomings clearly underlined and discussed in the kinetic analysis section, constitutes a useful tool to assess chemical pathways of fuel NO_*x*_ formation and to extend the fuel palette of the CRECK kinetic framework to include a representative compound to reproduce the N fuel content in FPBOs.

## 3 JSR Experiments

Different set of experiments have been performed covering both pyrolysis and oxidation conditions. The first section below describes the apparatus used to perform experiments as well as the analytical method. The second section describes the experimental data obtained for pyrrole pyrolysis and oxidation.

### 3.1 Experimental Method Description

The experimental setup of pyrrole was a laboratory-scale JSR (85 cm^3^) working close to atmospheric pressure (1.07 bar). This setup is described in detail elsewhere,^12,27,40^ and only a brief description is provided here. Experiments were performed in a fused silica JSR, a type of ideal continuously stirred-tank reactor, which is suitable for gas-phase kinetic studies. Reactants, with helium as the carrier gas, entered the spherical JSR through four nozzles located at its center, allowing for the creation of high turbulence resulting in homogeneity in temperature and composition. The reactor was heated using Inconel resistances, and the reaction temperature was measured with a K-type thermocouple positioned in a glass finger close to the center of the reactor (uncertainty of ±5 K). Pyrrole pyrolysis and oxidation under stoichiometric conditions were carried out at a residence time of about 2 s and at temperatures ranging from 700 to 1200 K with initial fuel mole fraction of ∼10 000 ppm. The experimental conditions are summarized in Table 2.

**Table 2:** Summary of JSR Experimental Conditions Used in the Present Study

					inlet mole fraction (%)		
set	*T* (K)	*p* (bar)	τ (s)^a^	φ^b^	pyrrole	O_2_	He
1	925–1200	1.067	2	∞	0.93	0.00	99.07
2	700–1200	1.067	2	0.5	1.05	12.95	86.00
3	700–1200	1.067	2	1	1.05	6.20	92.75
4	700–1200	1.067	2	2	1.05	3.08	95.87

a The residence time is defined as the ratio between the reactor volume and the gas flow rate (m^3^/s) under the conditions of the temperature and pressure in the reactor.

b The equivalence ratio was defined by considering the following stoichiometric equation: C_4_H_5_N + 6.25O_2_ → 4CO_2_ + 2.5H_2_O + NO_2_.

The purities of helium and oxygen were 99.99% and provided by Messer. Pyrrole was provided by Sigma-Aldrich with a claimed purity of 98% and used without further purification because a GC analysis did not identify any impurity in the reactant, despite the careful chromatogram analysis. A liquid Coriolis flow controller was used to control the flow of pyrrole, mixed with helium and passed through an evaporator (393 K) before being mixed with oxygen prior to entering the reactor. The relative uncertainty in gas flow rates is about 0.5%. Although the boiling point of pyrrole is ∼403 K, the temperature of the evaporator was set at a lower temperature to avoid the fouling and clogging of this part of the apparatus (it occurred several times, and the evaporator had to be cleaned; this operation was quite complex given the diameter of the tube inside the evaporator).

The reactants and reaction products leaving the reactors were then transported by a heated transfer line maintained at 393 K to avoid condensation to a Fourier transform infrared (FTIR) spectroscopy device and two gas chromatographs (GCs). The first GC, equipped with a Carbosphere-packed column, a thermal conductivity detector (TCD), and a flame ionization detector (FID), was used to quantify lightweight species. The second GC, fitted with a Q-Bond capillary column and a FID preceded by a methanizer, is used for the quantification of compounds containing two carbon atoms. The methanizer (nickel catalyst for hydrogenation) allows for the detection of species, like CO and CO_2_, and allows for the detection species, like CH_3_CHO, with better sensitivity. Moreover, FTIR spectroscopy is also used to quantify CO, CO_2_, and HCN. Identification of species was performed using a GC (with a Q-Bond capillary column) coupled to a mass spectrometer with electron impact ionization at 70 eV. The FTIR spectrometer from Thermo Scientific Antaris is equipped with a mercury cadmium telluride photoelectric detector. Spectra were recorded over the wavelength range of 400–4000 cm^–1^ with a resolution of 0.5 cm^–1^. The cell (optic path of 10 m) was heated to 373.15 K, and measurements were performed at a pressure of 150 Torr. An average of 32 scans was considered for a spectrum measurement. The detection limit depends upon the species, the absorption line used for the quantification, and possible interferences with other species. In the present study, it was about 35 ppm for HCN, 25 ppm for CO, and 2 ppm for CO_2_. Note that FTIR and GC analyses were performed in separated experiments. Excellent agreement was observed for carbon monoxide (a species with some isolated lines in the absorption spectra) mole fractions obtained with the two diagnostics.

GC calibrations were performed using gaseous standards provided by Messer and Air Liquide for small species, like carbon monoxide, carbon dioxide, oxygen, methane, and HCN. The calibration was performed for the fuel by injecting synthetic gas mixtures of pyrrole and helium. Other species detected with the FID were calibrated using the effective carbon number (ECN) method (their calibration factors were deduced from those of species calibrated taking into account their number of effective carbon atoms). FTIR calibrations were performed for all species, which were detected using this technique (CO, CO_2_, and HCN) using gaseous standards provided by Messer and Air Liquide. Relative uncertainties in mole fractions of species detected by GC and calibrated using gaseous standards provided by Messer and Air Liquide were estimated to ±5%. The relative uncertainty in the mole fractions of pyrrole was estimated to ±10%, although it was calibrated as a result of the difficulties in handling such a species. Relative uncertainties in mole fractions of species calibrated using the ECN methods were estimated to ±10%.

### 3.2 Experimental Results

Reaction products detected during the pyrolysis of pyrrole are hydrocarbons and N-containing species. Hydrocarbons are methane, acetylene, ethylene, ethane, propene, allene, propyne, a C_4_H_*x*_ species, which could not be clearly identified (but highly unsaturated), benzene, and toluene. N-containing species are hydrogen cyanide (HCN), acetonitrile (CH_3_CN), 2-propenenitrile (also called acrylonitrile, CH_2_CHCN), and three isomers for butenenitrile [but-3-enitrile, (2*E*)-but-2-enenitrile, and (2*Z*)-but-2-enenitrile, also named allyl cyanide, *trans*-crotonitrile, and *cis*-crotonitrile, respectively]. Note that the mass spectra for the three isomers are similar and that the peak attribution could not be performed with certainty. The conversion of pyrrole becomes significant from ∼1050 K. Mole fractions of most of species increase over the studied temperature range. The few species for which a maximum in mole fraction is observed are allene, propyne, propene, and all N-containing species, except HCN.

Reaction products detected during the oxidation experiments are the same as those detected during pyrolysis (except for toluene, only observed for pyrolysis). In addition, small oxygenated compounds, like carbon monoxide and carbon dioxide, are observed. The reactivity is enhanced in comparison to pyrolysis, with significant pyrrole conversions observed from ∼850 K. The equivalence ratio has an effect on the reactivity, with the leaner case being the most reactive and the richer case being the least reactive. At mid conversion, the three pyrrole mole fraction profiles are shifted by about 50 K under the conditions of the present study. In a general way, all detected intermediates see their mole fractions going through a maximum, except for carbon dioxide, which is an end product of combustion reactions. For the rich case, the deficit of O_2_ leads to higher mole fractions of intermediates and significant mole fractions of CO are still observed at 1200 K, which is the highest temperature considered in this study. For the stoichiometric and lean mixtures, some of the intermediates observed under pyrolysis and rich conditions are not detected (e.g., ethane, propene, allene, propyne, etc.).

Atomic balances were performed for all experiments. For pyrolysis, C, N, and H atomic balances are satisfactory up to 1100 K (they lie in between 1 and 0.85). Above 1100 K, the three atomic balances tend to decrease monotonously, reaching ∼0.5 for C and N and ∼0.7 for H. This was not surprising given the fouling that was observed in the lines between the outlet of the reactors and the analytical devices. The fouling is likely due to the condensation of some heavy aromatic species (possibly containing N atoms). For oxidation experiments, the carbon atom balance is satisfactory over the whole range of temperatures, usually lying in between 0.8 and 1, except in the range of 850–1000 K, where it falls to ∼0.8. In a general way, it is slightly better for the lean and stoichiometric mixtures than for the rich mixture. H and O atom balances are not meaningful because water, one of the main reaction products, was not quantified during this study. The N atomic balance is satisfactory up to 875 K, and then it decreases monotonically to 0 for the lean and stoichiometric cases and up to ∼0.4 for the rich case, indicating that one or several N-containing species were not detected during these oxidation experiments. The potential presence of some species, such as N_2_, NH_3_, NO, NO_2_, and N_2_O, was then further investigated. If present, N_2_ could be detected if above the detection limit (∼500 ppm) using GC with detection using the TCD. This species was not observed during oxidation nor during pyrolysis of pyrrole. If present in a sufficiently high concentration, NH_3_, NO, NO_2_, and N_2_O could be detected using the FTIR spectroscopy tool because they have very characteristic absorption structures in the wavenumber range investigated (detection limits of ∼2, ∼100, ∼10, and ∼50 ppm, respectively). None of these species could be identified in recorded spectra during oxidation experiments, even in traces.

## 4 Theoretical Methods for Thermodynamic Property Estimation

Thermodynamic properties of pyrrole, pyrrolenine, and pyrrolyl radical were calculated by first-principle calculations using the Gaussian 16 revision B suite of programs^41^ at the CBS-QB3^42^ and G4^43^ levels of theory as implemented. Both methods use B3LYP geometries and frequencies, although using different basis sets, and contain several energy calculation steps to extrapolate the electronic energy to a CCSD(T)/CBS level. Electronic energies are converted with the atomization method to the corresponding heats of formation. In the case of CBS-QB3, corrections for additive bond errors (BACs)^44^ were applied.

The thermal contributions to the enthalpy, the entropy at 298 K, and the heat capacities as a function of the temperature have been calculated with methods of statistical mechanics.^45^ The required input data (rotational constants, molecular weight, and scaled frequencies) are readily available from the CBS-QB3 calculations. The harmonic oscillator, rigid rotor assumption is applied because the species of interest do not contain internal rotations. The calculated total entropies contain corrections for the symmetry if needed. The thermodynamic data are converted to NASA polynomials and used in the kinetic model.

The National Institute of Standards and Technology (NIST) WebBook contains two very different entries for the enthalpy of formation of pyrrole: Δ_f_*H*^298^ = 143.2 kJ/mol^46^ and 108.3 kJ/mol.^47^ The current CBS-QB3 result of Δ_f_*H*^298^ = 106.1 kJ/mol agrees well with the older experimental value. With G4, a Δ_f_*H*^298^ value of 109.7 kJ/mol is obtained, which supports CBS-QB3 as well as the lower experimental enthalpies of formation. The data are also in agreement with the previous works by Simmie,^48^ who reports 109.2 ± 2.3 kJ/mol through isodesmic reactions and 110.9 kJ/mol via the atomization method, and Lo and Lau,^49^ who report Δ_f_*H*^298^ = 110.9 kJ/mol using a CCSD(T)/CBS approach.

## 5 Kinetic Model

The kinetic model to describe pyrrole pyrolysis and oxidation accounts for 189 chemical species and 2888 reactions and is available as Supporting Information together with thermodynamic properties (model 1). Specifically, the pyrrole pyrolysis and oxidation subset contains 33 species and 456 reactions. Chemical structures and names of relevant chemical species in the pyrrole kinetic subset are reported in Table 1 (section 2).

The kinetic model builds on the CRECK core mechanism composed of a hydrogen subset by Kéromnés et al.,^50^ C_1_–C_2_ from Metcalfe et al.,^51^ and C_3_ and molecular growth pathways from Burke et al.^52^ and Ranzi et al.,^53,54^ recently updated by Bagheri et al.^55^ A NO_*x*_ kinetic subset is adopted from Song et al.,^27^ with small updates concerning acetonitrile (CH_3_CN) from the recent study by Alzueta et al.^56^ Aiming for a hierarchical development of the CRECK kinetic modeling framework, modifications to relevant kinetic subsets, such as those describing NO_*x*_ or the core C_0_–C_4_ mechanism that have already been addressed in recent efforts,^27,55^ are outside the scope of this study. Thermodynamic properties of relevant species have been determined as described in section 4 or taken from previous studies.^27,32,57−59^Table 3 lists important reactions in the pyrrole pyrolysis and oxidation subset, with detailed references to the source of selected rate coefficients and notes on minor modifications applied for improved agreement based on insights gained from the kinetic analysis below. Sources of rate constants for remaining reactions of the pyrrole/pyrrolenine subset not reported in Table 3 are provided in detail in the Supporting Information.

**Table 3:** Rate Coefficients for Relevant Reactions in the Following Discussion on Pyrrole Pyrolysis and Oxidation^a^

	reaction	*A*	*n*	*E*_a_	reference	notes
R1	C_4_H_5_N ↔ PYRLNE	3.16 × 10^13^	0.005	46300	(32)	
R2	C_4_H_5_N ↔ HNCPROP	1.10 × 10^14^	0.000	77162	(32)	
R3	PYRLNE ↔ aC_3_H_5_CN	5.24 × 10^15^	0.000	75710	(32)	*A* × 2
R4	PYRLNE ↔ *c*C_3_H_5_CN	1.65 × 10^15^	0.000	70050	(32)	*A*/2
R5	PYRLNE ↔ HNCPROP	2.51 × 10^15^	0.000	79474	(32)	
R6	HNCPROP ↔ HCN + C_3_H_4_-p	5.50 × 10^12^	0.000	37740	(32)	*A*/2, *E*_a_ = +1500 cal/mol
R7	PYRLNE ↔ Ḣ + aĊ_3_H_4_CN	2.04 × 10^17^	0.000	86746	(32)	*A* × 2
R8	aC_3_H_5_CN ↔ *t*C_3_H_5_CN	7.00 × 10^14^	0.000	61969	(32)	
R9	aC_3_H_5_CN ↔ *c*C_3_H_5_CN	7.20 × 10^14^	0.000	58863	(32)	
R10	*c*C_3_H_5_CN ↔ *t*C_3_H_5_CN	1.40 × 10^14^	0.000	57573	(32)	
R11*	aC_3_H_5_CN ↔ Ċ_2_H_3_ + ĊH_2_CN	3.40 × 10^15^	0.000	82640	pw	*C_4_H_8_-1 ↔ C_2_H_3_ + C_2_H_5_
R12	aC_3_H_5_CN + Ḣ ↔ C_2_H_4_ + ĊH_2_CN	1.00 × 10^13^	0.000	3010	(57)	*A* × 2
R13	*t*C_3_H_5_CN + Ḣ ↔ HCN + Ċ_3_H_5_-s	6.00 × 10^12^	0.000	4000	pw	*H + C_2_H_2_
R14	Ḣ + C_4_H_5_N ↔ H_2_ + PYRLYL	1.00 × 10^6^	2.000	2825	pw	*H-abs. tertiary C–H
R15	ĊH_3_ + C_4_H_5_N ↔ CH_4_ + PYRLYL	4.50 × 10^4^	2.000	3778	pw	
R16	ĊH_2_CN + C_4_H_5_N ↔ CH_3_CN + PYRLYL	1.35 × 10^4^	2.000	12460	pw	*C_3_H_3_ + C_4_H_5_N
R17	ȮH + C_4_H_5_N ↔ H_2_O + PYRLYL	9.00 × 10^8^	1.000	–695	pw	
R18	HȮ_2_ + C_4_H_5_N ↔ H_2_O_2_ + PYRLYL	3.60 × 10^6^	2.000	14440	pw	
R19	O_2_ + C_4_H_5_N ↔ HȮ_2_ + PYRLYL	8.00 × 10^13^	0.000	37150	pw	
R20	Ö + C_4_H_5_N ↔ ȮH + PYRLYL	1.10 × 10^6^	2.000	1404	pw	
R21	Ḣ + aC_3_H_5_CN ↔ H_2_ + aĊ_3_H_4_CN	1.90 × 10^2^	3.500	1627	pw	*R + C_4_H_8_-1 ↔ C_4_H_7_1–3 + RH (*A*/3)
R22	ĊH_3_ + aC_3_H_5_CN ↔ CH_4_ + aĊ_3_H_4_CN	7.14 × 10^0^	3.500	7642	pw	
R23	ĊH_2_CN + aC_3_H_5_CN ↔ CH_3_CN + aĊ_3_H_4_CN	2.00 × 10^11^	0.000	12000	(57)	*A*/2
R24	ȮH + aC_3_H_5_CN ↔ H_2_O + aĊ_3_H_4_CN	7.70 × 10^5^	2.200	–437	pw	
R25	HȮ_2_ + aC_3_H_5_CN ↔ H_2_O_2_ + aĊ_3_H_4_CN	7.82 × 10^–1^	3.970	11702	pw	
R26	O_2_ + aC_3_H_5_CN ↔ HȮ_2_ + aĊ_3_H_4_CN	5.00 × 10^13^	0.000	37190	pw	
R27	Ö + aC_3_H_5_CN ↔ ȮH + aĊ_3_H_4_CN	1.75 × 10^11^	0.700	5884	pw	
R28	Ḣ + *t*C_3_H_5_CN ↔ H_2_ + aĊ_3_H_4_CN	3.64 × 10^5^	2.455	4361	pw	*R + C_3_H_6_=C_3_H_5_-A + RH
R29	ĊH_3_ + *t*C_3_H_5_CN ↔ CH_4_ + aĊ_3_H_4_CN	2.21 × 10^0^	3.500	5675	pw	
R30	ĊH_2_CN + *t*C_3_H_5_CN ↔ CH_3_CN + aĊ_3_H_4_CN	5.00 × 10^12^	0.000	10989	(57)	*A*/2
R31	ȮH + *t*C_3_H_5_CN ↔ H_2_O + aĊ_3_H_4_CN	4.46 × 10^6^	2.072	1051	pw	*R + C_3_H_6_=C_3_H_5_-A + RH
R32	HȮ_2_ + *t*C_3_H_5_CN ↔ H_2_O_2_ + aĊ_3_H_4_CN	3.07 × 10^–2^	4.403	13547	pw	
R33	O_2_ + *t*C_3_H_5_CN ↔ HȮ_2_ + aĊ_3_H_4_CN	1.20 × 10^20^	–1.67	46191	pw	
R34	Ö + *t*C_3_H_5_CN ↔ ȮH + aĊ_3_H_4_CN	5.24 × 10^11^	0.700	5884	pw	
R35	Ḣ + *t*C_3_H_5_CN ↔ H_2_ + Ċ_3_H_4_CN	2.25 × 10^7^	1.930	12950	pw	*R + C_2_H_4_=C_2_H_3_ + RH (*A*/2)
R36	ĊH_3_ + *t*C_3_H_5_CN ↔ CH_4_ + Ċ_3_H_4_CN	4.85 × 10^2^	2.947	15148	pw	
R37	ĊH_2_CN + *t*C_3_H_5_CN ↔ CH_3_CN + Ċ_3_H_4_CN	5.00 × 10^12^	0.00	12000	(57)	*A*/2
R38	ȮH + *t*C_3_H_5_CN ↔ H_2_O + Ċ_3_H_4_CN	1.11 × 10^4^	2.745	2216	pw	*R + C_2_H_4_=C_2_H_3_ + RH (*A*/2)
R39	HȮ_2_ + *t*C_3_H_5_CN ↔ H_2_O_2_ + Ċ_3_H_4_CN	2.15 × 10^5^	2.000	20243	pw	
R40	O_2_ + *t*C_3_H_5_CN ↔ HȮ_2_ + Ċ_3_H_4_CN	2.11 × 10^13^	0.000	57623	pw	
R41	Ö + *t*C_3_H_5_CN ↔ ȮH + Ċ_3_H_4_CN	1.08 × 10^7^	2.000	8782	pw	
R42	aĊ_3_H_4_CN ↔ *c*Ċ_3_H_4_CN	5.00 × 10^13^	0.000	51983	(57)	
R43	PYRLYL ↔ *c*Ċ_3_H_4_CN	1.50 × 10^13^	0.000	38987	(32)	
R45	Ċ_3_H_4_CN ↔ ĊH_3_ + C_3_HN	6.00 × 10^14^	0.000	42000	(57)	
R46	*c*Ċ_3_H_4_CN ↔ C_2_H_2_ + ĊH_2_CN	1.07 × 10^15^	–0.560	36320	pw	*C_4_H_7_1–1 ↔ C_2_H_2_ + C_2_H_5_
R47	Ḣ + C_3_HN ↔ C_2_H_2_ + ĊN	1.00 × 10^14^	0.000	2000	pw	*H + C_2_H_2_
R48	aĊ_3_H_4_CN + HȮ_2_ ↔ C_4_H_4_NȮ + ȮH	1.95 × 10^18^	–1.060	7852	pw	*C_3_H_5_-A + HO_2_ (1 atm)
R49	cĊ_3_H_4_CN + O_2_ ↔ C_4_H_4_NȮ + Ö	2.30 × 10^20^	–2.650	6489	pw	*C_3_H_5_-A + O_2_ (1 atm)
R50	C_4_H_4_NȮ ↔ C_2_H_3_CHO + ĊN	1.50 × 10^13^	0.000	33000	(60)	C–C β-scission
R51	C_4_H_4_NȮ → C_2_H_2_ + CH_2_O + ĊN	1.50 × 10^13^	0.000	33000	(60)	C–C β-scission
R52	ĊH_2_CN + ĊH_2_CN ↔ C_4_H_4_N_2_	2.30 × 10^13^	0.000	0.000	(58)	
R53	CH_3_CN (+M) ↔ ĊH_2_CN + H (+M)	9.20 × 10^12^	0.850	95770	(58)	high-pressure limit
R54	ĊH_2_CN + C_4_H_4_N_2_ ↔ CH_3_CN + Ċ_4_H_3_N_2_	3.50 × 10^12^	0.000	5000	(58)	
R55	Ċ_4_H_3_N_2_ ↔ CH_2_CHCN + ĊN	4.40 × 10^14^	0.000	55000	(58)	
R56	NO + Ö (+M) ↔ NO_2_ (+M)	1.30 × 10^15^	–0.750	0.000	(61)	
R57	Ö + C_3_HN ↔ CO + H*C̈*–CN	7.40 × 10^8^	1.280	2472	pw	*C_2_H_2_ + O ↔ CH_2_ + CO
R58	O_2_ + H*C̈*–CN ↔ CO_2_ + HCN	1.10 × 10^12^	0.000	0.000	(56)	
R59	ȮH + CH_3_CN ↔ H_2_O + ĊH_2_CN	2.00 × 10^7^	2.000	5000	pw	*OH + C_3_H_4_-p ↔ C_3_H_3_ + H_2_O
R60	ĊH_2_CN + Ö ↔ Ḣ + OCHCN	3.00 × 10^11^	0.640	0.000	(56)	
R61	OCHCN ↔ HCN + CO	3.50 × 10^14^	0.000	66300	(62)	
R62	ȮH + HCN ↔ Ḣ + HNCO	1.71 × 10^11^	0.000	8744	(63)	
R63	ȮH + HCN ↔ H_2_O + ĊN	1.45 × 10^13^	0.000	10900	(64)	
R64	ȮH + HNCO ↔ H_2_O + ṄCO	3.50 × 10^6^	1.500	3600	(65)	
R65	O_2_ + ṄCO ↔ CO_2_ + NO	2.00 × 10^12^	0.000	20000	(66)	
R66	C_2_N_2_ + M ↔ ĊN + ĊN + M	1.60 × 10^34^	–4.32	130000	(67)	
R67	C_2_N_2_ + Ḣ ↔ HCN + ĊN	3.10 × 10^14^	0.000	7860	(58)	
R68	HĊCO + NO ↔ HCN + CO_2_	2.23 × 10^14^	–0.750	400	(68)	

a Rate coefficients refer to an Arrhenius expression of the rate constants as *k* = *AT*^*n*^ exp(−*E*_a_/*RT*). Units are cal, mol, K, cm, and s. Reactions noted as “pw” have been estimated in the present work. Analogy assumptions are noted with an asterisk.

## 6 Results and Discussion

Results from model simulations are compared to the new JSR data discussed in section 3 as well as other targets from the literature.^29,35,36,39^ Kinetic analyses and discussion highlight relevant reaction pathways for both pyrolysis (section 6.1) and oxidation conditions (section 6.2).

### 6.1 Pyrolysis

**Figure 1:**
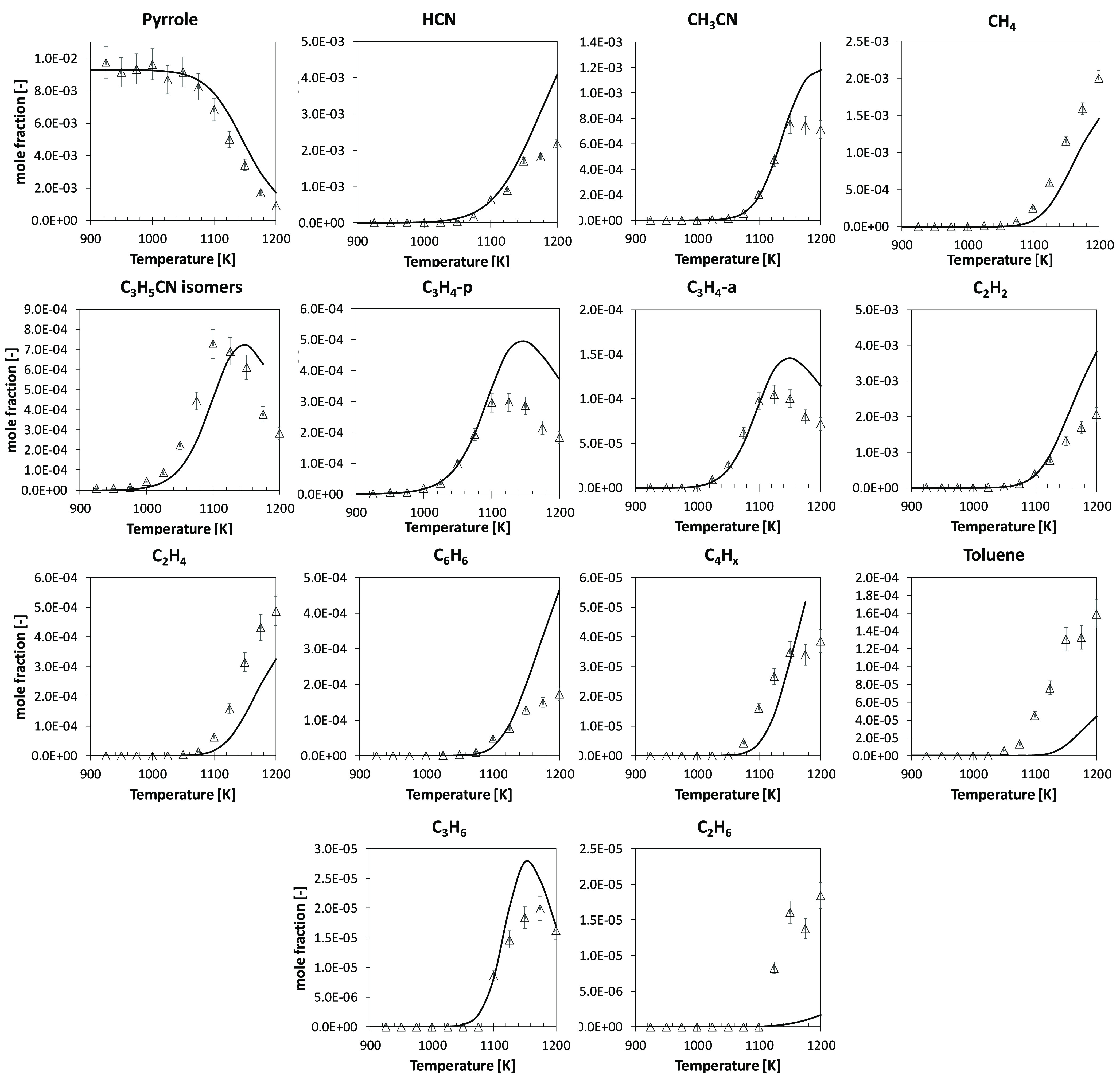
Pyrolysis of pyrrole (∼1 mol % in helium) in a JSR at *p* = 107 kPa and τ = 2.0 s. Comparison between experimental (symbols) and predicted (lines) mole fraction profiles of intermediate and product species.

Figure 1 compares model predictions to the newly acquired pyrolysis data in a JSR, operating at *p* = 107 kPa and τ = 2.0 s in the temperature range *T* = 900–1200 K. Good agreement is observed for pyrrole conversion, in particular concerning the temperature of onset of reactivity. Pyrrole consumption is slightly underestimated for *T* > 1100 K. Molecular nitrogen is mainly converted into HCN, CH_3_CN, and C_3_H_5_CN isomers, i.e., *cis*- and *trans*-crotonitrile and allyl cyanide.

**Figure 2:**
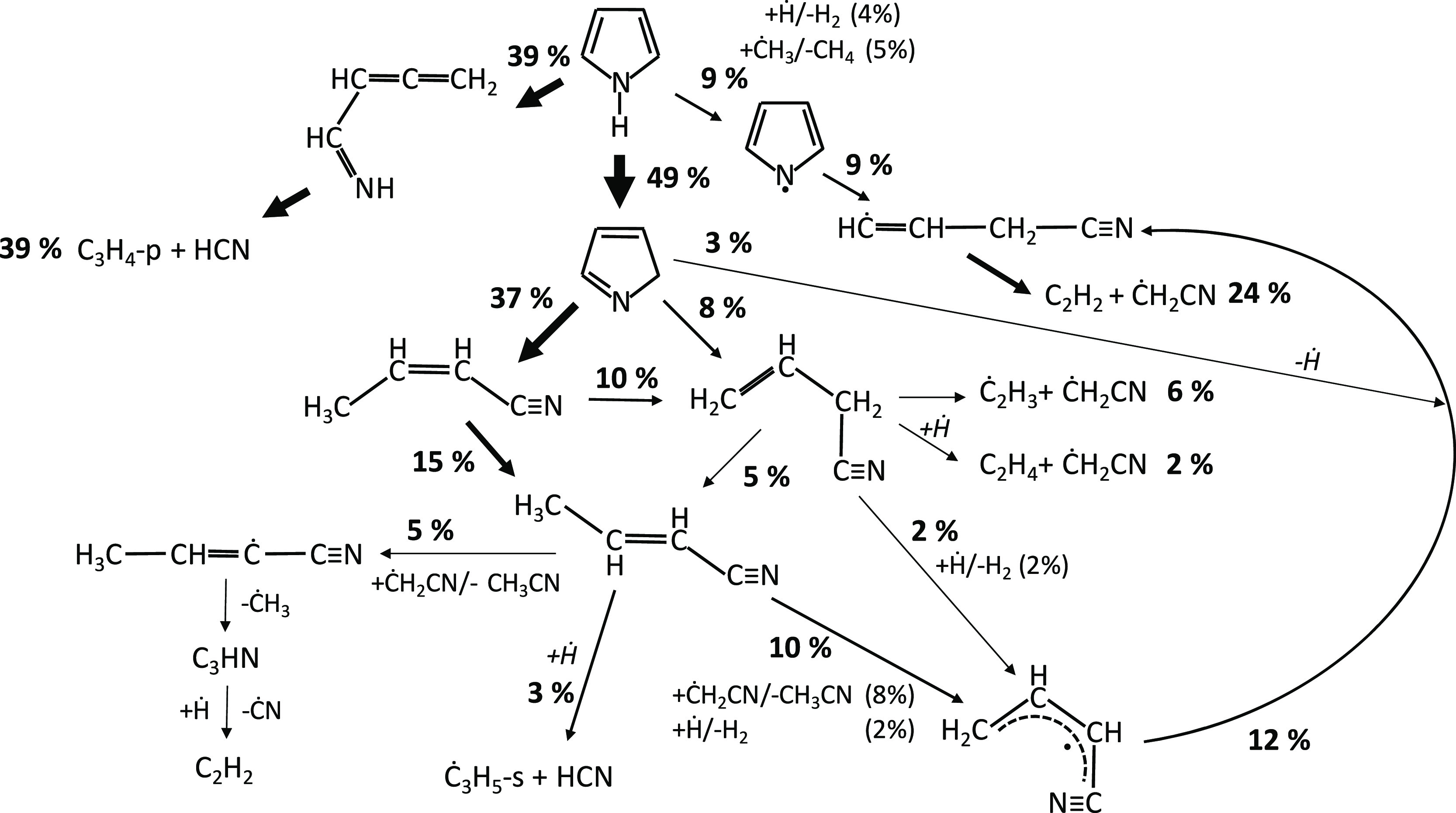
Rate of production analysis at *T* = 1100 K for a pyrrole/helium (1/99 mol %) mixture at *p* = 107 kPa and τ = 2.0 s. Arrow width qualitatively represents the importance of each reactive flux. Pathways with a flux going from or to an intermediate of <1% have been disregarded for clarity.

**Figure 3:**
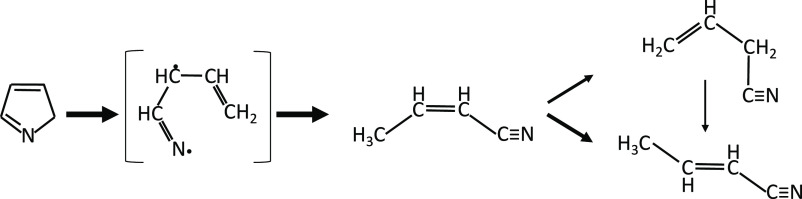
Ring-opening reaction of pyrrolenine to form crotonitrile isomers and allyl cyanide (C_3_H_5_CN isomers) through a biradical intermediate.

Figure 2 reports results from a rate of production analysis carried out at *T* = 1100 K for the JSR experiments of Figure 1. Pyrrole is largely consumed by the isomerization to pyrrolenine (reaction R1), followed by the isomerization to allenic imine (reaction R5). This latter step occurs through a 1,4-H migration of the biradical intermediate, resulting from the ring-opening reaction of pyrrolenine (Figure 3), as discussed by Martoprawiro et al.^32^

The same intermediate leads to the formation of allyl cyanide and crotonitrile tautomers from pyrrolenine (reactions R3 and R4). For these pathways, we adopted the high-pressure limit rate coefficients from Martoprawiro et al.^32^ as a result of the lack of a systematic investigation of the pressure dependence in the literature. Pyrrolenine preferentially forms *cis*-crotonitrile (reaction R4) that further isomerizes to allyl cyanide (reaction R9) or tautomerizes to *trans*-crotonitrile (reaction R10), as shown in Figure 3.

**Figure 4:**
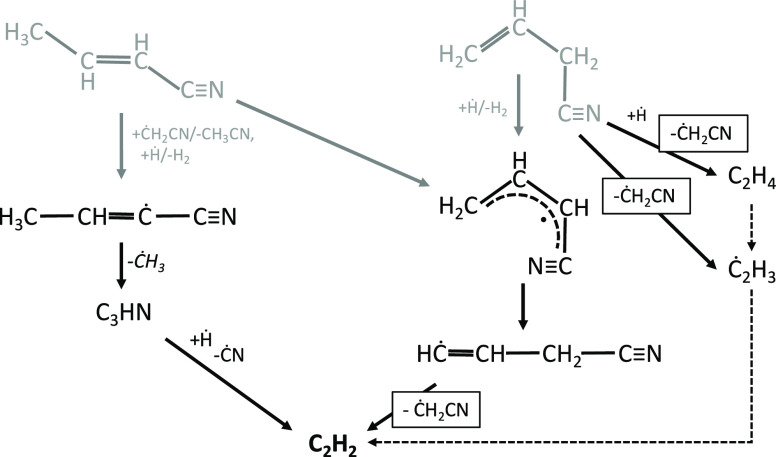
Pathways leading to the formation of acetylene and the cyanomethyl radical in pyrrole pyrolysis.

H-abstractions by Ḣ and ĊH_2_CN from C_3_H_5_CN isomers (reactions R21, R23, R28, and R30) lead to the formation of the cyanoallyl radical (aĊ_3_H_4_CN) or to its non-allylic isomer (*c*Ċ_3_H_4_CN) as well as H_2_ and acetonitrile (CH_3_CN). *c*Ċ_3_H_4_CN completely decomposes to methyl radical (ĊH_3_) and cyanoacetylene (C_3_HN) through reaction R45. aĊ_3_H_4_CN can further isomerize to another non-allylic isomer (Ċ_3_H_4_CN) through a 1,3-H migration (reaction R42). This latter intermediate undergoes a β-scission reaction (reaction R46), forming acetylene and cyanomethyl radical (ĊH_2_CN). This pathway constitutes a major source of ĊH_2_CN, together with the unimolecular decomposition reaction and the H-addition/decomposition reaction of allyl cyanide, forming vinyl radical (Ċ_2_H_3_) and ethylene (C_2_H_4_). The reaction pathways discussed above ultimately lead to the formation of acetylene, as summarized in Figure 4. The cyanomethyl radical (ĊH_2_CN) produced by the decomposition channels depicted in Figure 4 is transformed to acetonitrile through H-abstraction reactions (reactions R16, R23, R30, R37, etc.).

Allenic imine is entirely converted into propyne and hydrogen cyanide (HCN) through reaction R6. For this channel, we adopted the rate constant suggested by Martoprawiro et al.,^32^ with some correction, as reported in Table 3, to better match C_3_H_4_-p and HCN profiles in pyrolysis and oxidation experiments. Our modifications are within the uncertainties discussed in ref (32), i.e., 1–2 kcal/mol in single-point energy calculations.

Allene is formed by the isomerization reaction C_3_H_4_-p ↔ C_3_H_4_-a and consumed by molecular growth pathways, leading to cyclopentadiene (C_2_H_2_ + C_3_H_4_-a ↔ C_5_H_6_) and benzene (Ċ_3_H_3_ + C_3_H_4_-a ↔ C_6_H_6_ + Ḣ). C_4_H_*x*_ represents the sum of 1,2-butadiene (C_4_H_6_) and but-1-en-3-yne (C_4_H_4_), whose peaks were too close to be distinguished in the experimental measurements. Propyne and allene reactions with the methyl radical produce butadiene (ĊH_3_ + C_3_H_4_-p ↔ Ḣ + C_4_H_6_ and ĊH_3_ + C_3_H_4_-a ↔ Ḣ + C_4_H_6_), while self-recombination reactions produce but-1-en-3-yne (C_3_H_4_-p + C_3_H_4_-p → C_2_H_4_ + C_4_H_4_ and C_3_H_4_-a + C_3_H_4_-a → C_2_H_4_ + C_4_H_4_). H-abstraction reactions by Ḣ and ĊH_3_ also contribute to fuel consumption, forming the resonance-stabilized pyrrolyl radical. On the basis of the dissociation energy of the N–H bond (BDE_298 K_ = 96.1 kcal/mol), for H-abstraction reactions leading to the pyrrolyl radical, we adopted the values for the H-abstractions of a tertiary H atom according to the generalized approach by Ranzi et al.^69^ The pyrrolyl radical entirely isomerizes to *c*Ċ_3_H_4_CN (reaction R43), further contributing to acetylene and ĊH_2_CN production.

**Figure 5:**
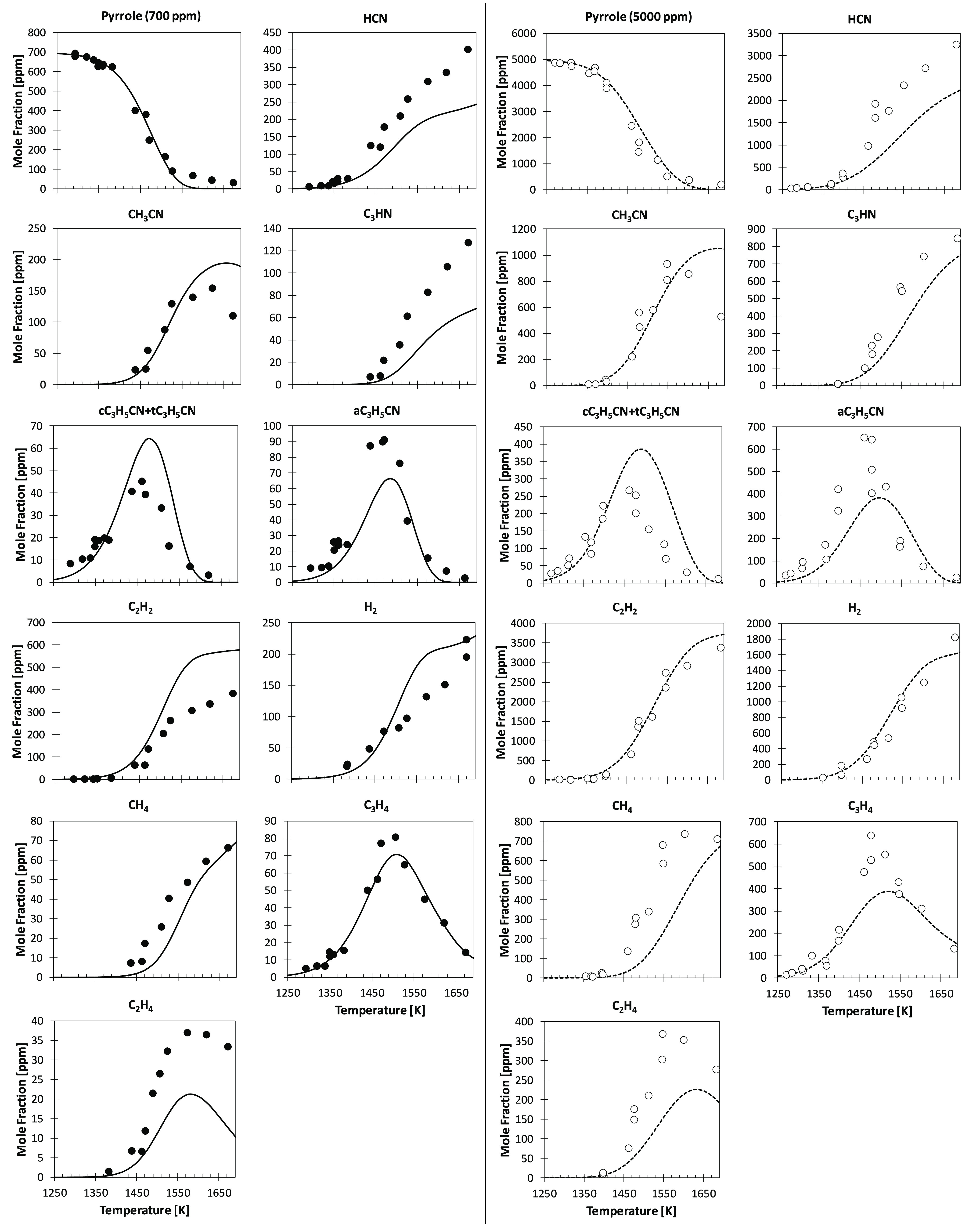
Pyrolysis of pyrrole (700 ppm, left panel, and 5000 ppm, right panel, in argon) in a single-pulse shock tube at *p* = 13 atm and τ = 550 μs. Comparison between experimental (symbols)^29^ and predicted (lines) mole fraction profiles of intermediate and product species.

Figure 5 compares model results to the single-pulse shock-tube data by Mackie et al.^29^ Fuel conversion profiles are well-captured by the model for both the 700 and 5000 ppm cases.

**Figure 6:**
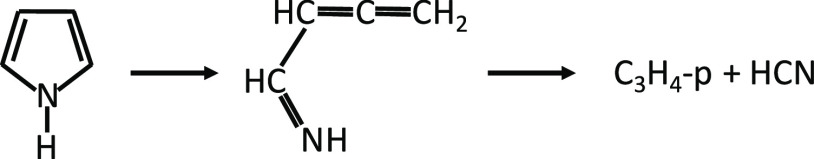
Pyrrole isomerization to allenic imine (reaction R2) HNCPROP and successive unimolecular decomposition to hydrogen cyanide and propyne (reaction R6).

HCN that was overestimated in JSR experiments (Figure 1) is now underestimated by a similar extent, i.e., factor of ∼2. The decomposition of allenic imine is still the major source of HCN (Figure 6), thus preventing any optimization of the rate coefficients for this reaction in one direction or the other.

**Figure 7:**
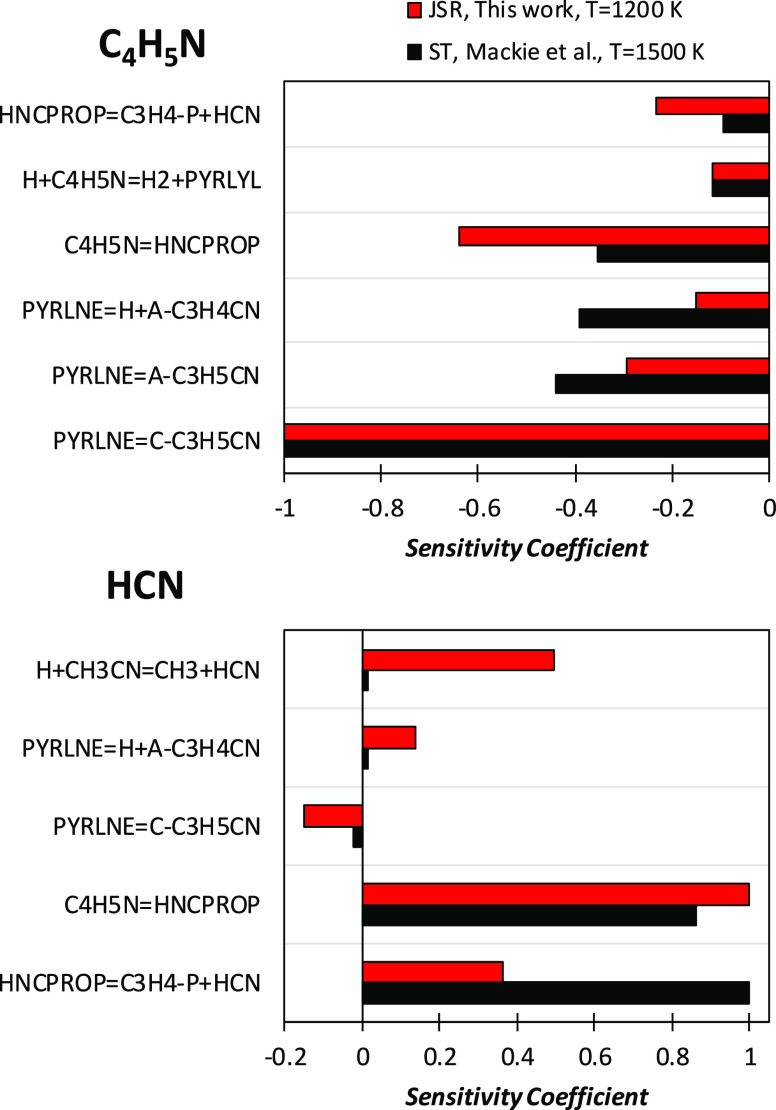
Sensitivity analysis of fuel consumption (top panel) and HCN formation (bottom panel) to model rate constants for the JSR case (Figure 1; *T* = 1200 K) and the ST case (Figure 5; 5000 ppm of pyrrole and *T* = 1500 K).

To further highlight the need for reconciling model and experiments for pyrrole pyrolysis and to support the modification of the rate constants for reaction R6, we performed a sensitivity analysis to highlight dominant reaction channels in both shock-tube (ST) experiments and our new JSR measurements. For the ST experiments, we performed a sensitivity analysis at *T* = 1500 K, corresponding to 80% fuel conversion. A similar conversion is obtained in the JSR experiments at *T* = 1200 K. Figure 7 shows the sensitivity coefficients of the most sensitive reactions controlling pyrrole consumption (top panel) and HCN formation (bottom panel). Concerning JSR simulations, pyrrole consumption is dominated by the isomerization reactions of pyrrolenine to *cis*-crotonitrile and allyl cyanide (reactions R3 and R4), together with pyrrole isomerization to allenic imine (reaction R2). The same subset of reactions controls fuel conversion in shock-tube pyrolysis. To a minor extent, H-abstractions by Ḣ and the decomposition of allenic imine to HCN and propyne (reaction R6) also influence pyrrole consumption. HCN formation is dominated by the reaction series R2 > R6 depicted in Figure 6 in both JSR and ST cases. However, for the JSR case, HCN formation is highly sensitive to successive isomerization and decomposition reactions of pyrrolenine (reactions R4 and R7) and mostly to Ḣ ipso-addition reaction of acetonitrile, Ḣ + CH_3_CN ↔ ĊH_3_ + HCN. Overall, despite quite different operating conditions, no reactions with opposite effects on model predictions for HCN emerged from this analysis, hampering improved agreement for both JSR and ST pyrolysis data.

The model is once again able to properly predict formation and consumption of C_3_H_5_CN isomers, which in Figure 5 are resolved in crotonitrile isomers and allyl cyanide. Acetonitrile formation is very well-captured; however, the model underpredicts its consumption at the higher temperature end of both the shock-tube (*T* > 1600 K) and JSR (*T* > 1150 K) experiments. As previously discussed, we adopted rate coefficients for the major consumption pathways of CH_3_CN from the recent study by Alzueta et al.^56^ C_2_H_2_ is also formed in this case by the decomposition reaction of *c*Ċ_3_H4CN (Figure 4), for which no theoretical estimates exist. We estimated a value by analogy starting from the decomposition of the vinyl radical of 1-butene (Ċ_4_H_7_1–1 ↔ C_2_H_2_ + Ċ_2_H_5_) and increasing its activation energy by 6 kcal/mol to better match acetylene and acetonitrile profiles in both pyrolysis and oxidation experiments. Methane and hydrogen are produced by H-abstraction reactions from the fuel, crotonitrile isomers, and propyne. Good agreement is observed for H_2_, while methane is slightly underestimated in both Figures 3 and 5.

### 6.2 Oxidation

**Figure 8:**
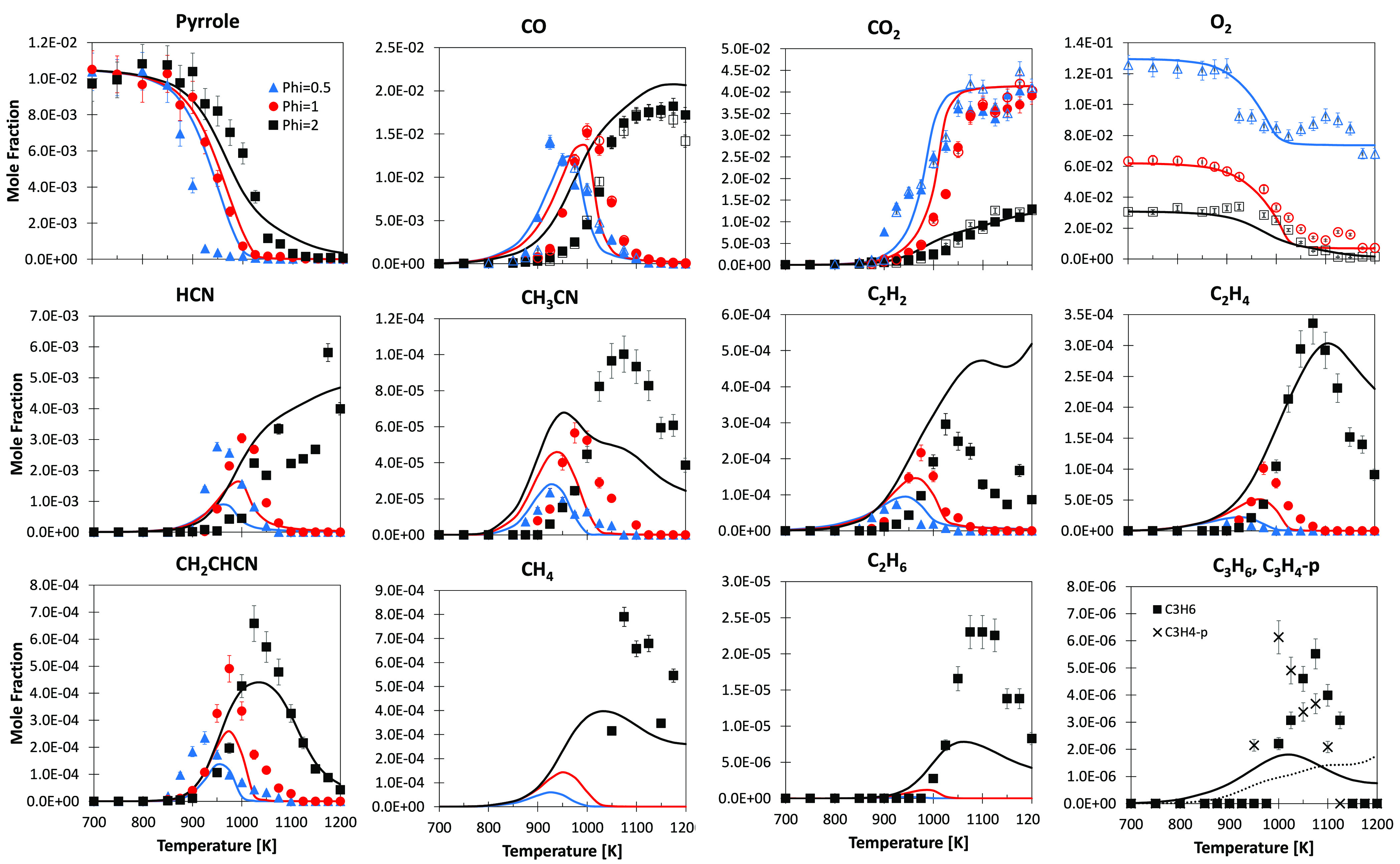
Pyrrole oxidation in JSR at φ = 0.5 (blue), φ = 1.0 (red), and φ = 2.0 (black), *p* = 107 kPa, and τ = 2.0 s. Comparison between experimental (symbols) and predicted (lines) fuel conversion and mole fraction profiles for intermediate and product species. (Open symbols) GC–TCD–FID with a Carbosphere-packed column and (full symbols) GC–methanizer–FID with a Q-Bond capillary column.

Experimental measurements and model predictions for the oxidation of pyrrole in a JSR are reported in Figure 8 for three equivalence ratios φ = 0.5, 1.0, and 2.0. Fuel consumption is correctly predicted by the model for the φ = 1.0 case. For the leanest case (φ = 0.5), the model strongly underpredicts fuel reactivity, despite correctly capturing the onset of conversion. In the richest case (φ = 2.0), the model captures the start of reactivity, slightly overpredicting pyrrole consumption for *T* < 1050 K and slightly underpredicting the complete conversion at higher temperatures.

Major product formation, such as CO and CO_2_, and O_2_ consumption are correctly reproduced, increasing confidence in the model’s validity. The effect of the equivalence ratio is qualitatively captured for all of the intermediates, but some major deviation in quantitative terms is observed. HCN formation is underestimated by ∼35% for the lean and stoichiometric cases but is overestimated for the rich case. Good agreement is observed in the case of CH_3_CN, with the exception of the rich case, where the model predicts an excessive consumption, underestimating the peak concentration by a factor of ∼2. Similar deviations are observed for acetylene, while ethylene peaks are quite nicely reproduced. The acrylonitrile (CH_2_CHCN) peak is underestimated at every equivalence ratio, and a delayed formation is observed at φ = 0.5 because of the underestimation of fuel reactivity in such conditions. Despite methane only being detected for the rich case, the model predicts its formation in significant quantities also for the lean and stoichiometric cases (i.e., 80–200 ppm). Ethane, propene, and propyne are only detected in very low quantities (i.e., 10–20 ppm) and only at φ = 2.0 in the experimental measurements. The model generally underestimates these minor products.

**Figure 9:**
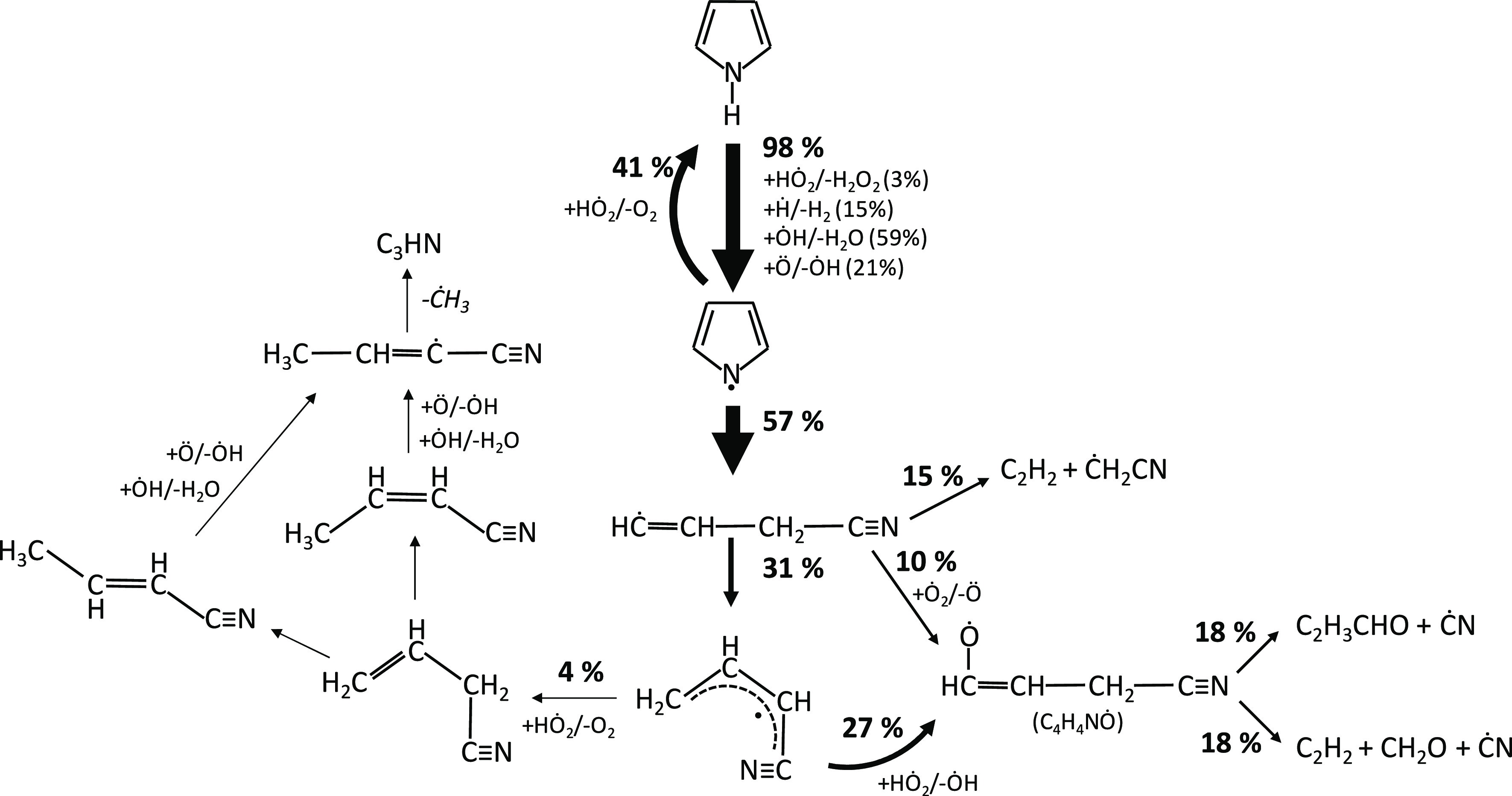
Rate of production analysis at *T* = 950 K for pyrrole oxidation in JSR at φ = 1.0, *p* = 107 kPa, and τ = 2.0 s. Arrow width qualitatively represents the importance of each reactive flux. Pathways with a flux going from or to an intermediate of <1% have been disregarded for clarity.

Figure 9 shows the rate of production analysis at *T* = 950 K for the stoichiometric case of Figure 8 (φ = 1.0). Pyrrole is consumed through H-abstraction reactions by ȮH, Ö, Ḣ, and HȮ_2_ to form the pyrrolyl radical that is largely converted back to pyrrole through the reverse reaction C_4_H_5_N + O_2_ ↔ PYRLYL + HȮ_2_ (reaction R19). This reaction is highly endothermic (*E*_a_ = 46.9 kcal/mol) and most likely proceeds in the backward direction at conditions where the HȮ_2_ concentration is high (e.g., *T* < 1000 K), as observed in the same system for other fuels too.^70^ This channel contributes to 41% of pyrrolyl consumption, while its decomposition to *c*Ċ_3_H_4_CN (reaction R43) accounts for 57% of the total flux. At such low temperatures, isomerization of *c*Ċ_3_H_4_CN to aĊ_3_H_4_CN (cyano allyl radical) through reaction R42 dominates over decomposition pathways, forming acetylene and ĊH_2_CN (reaction R46). aĊ_3_H_4_CN reacts with HȮ_2_ in reaction R48, releasing ȮH and forming a cyano alkoxy radical (C_4_H_4_NȮ) that decomposes through β-scission to form a cyano radical (ĊN) and unsaturated products, such as acrolein (C_2_H_3_CHO), or acetylene and formaldehyde (reactions R50 and R51). To a lower extent, a recombination/disproportionation reaction with HȮ_2_ can also occur, forming allyl cyanide and O_2_ (reaction R26), activating the isomerization reactions of C_3_H_5_CN isomers, until H-abstraction reactions by Ö form Ċ_3_H_4_CN (e.g., reaction R41) that further decomposes to the methyl radical and cyanoacetylene through β-scission (reaction R45). The rate coefficients for these pathways were estimated on the basis of analogy with the allyl radical (Ċ_3_H_5_-a)/HȮ_2_ kinetics.

The same cyano alkoxy radical can be formed directly from the interaction of *c*Ċ_3_H_4_CN with O_2_ (reaction R42), releasing Ö atoms. The rate coefficients for this channel have been adopted in analogy with Ċ_2_H_3_ + O_2_.

**Figure 10:**
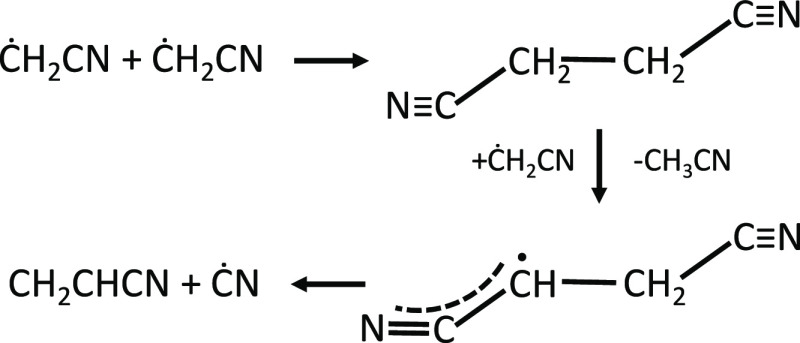
Succinonitrile (C_4_H_4_N_2_) formation and consumption pathways in pyrrole oxidation.

HCN is one of the main intermediates in pyrrole pyrolysis and oxidation. Different from the pyrolysis cases discussed above, where the main source was the decomposition of the allenic imine HNCPROP, at *T* = 950 K and in the presence of oxygen, hydrogen cyanide is mostly formed by the reaction of the isocyanate radical (ṄCO) with acetylene, forming HĊCO (C_2_H_2_ + ṄCO ↔ HĊCO + HCN). Acetonitrile is mainly formed by H-abstraction reactions of ĊH_2_CN on succinonitrile (C_4_H_4_N_2_, CN–CH_2_–CH_2_–CN). Succinonitrile (butanedinitrile) is formed by the self-recombination of ĊH_2_CN (reaction R52). H-abstraction reactions on C_4_H_4_N_2_, for example by ĊH_2_CN (reaction R54), produce a resonance-stabilized Ċ_4_H_3_N_2_ radical, whose decomposition reaction justifies the formation of acrylonitrile (Ċ_4_H_3_N ↔ CH_2_CHCN + ĊN, reaction R55) as illustrated in Figure 10.

For the succinonitrile subset, we entirely adopted the values proposed by Sendt et al.^58^ and assigned H-abstraction rate coefficients based on analogy with R + C_4_H_8_-1 = RH + C_4_H_7_1–3, accounting for the availability of four H atoms to form a resonance-stabilized radical rather than two, as in the case of 1-butene.

**Figure 11:**
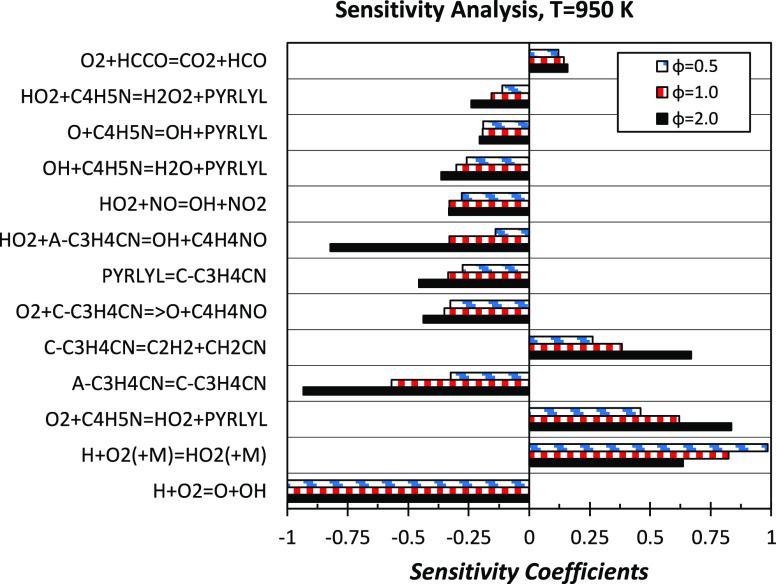
Sensitivity analysis of fuel consumption to model rate constants at *T* = 950 K for the φ = 0.5, 1.0, and 2.0 mixtures. Sensitivity coefficients are normalized over that of Ḣ + O_2_ ↔ Ö + ȮH. A negative sensitivity coefficient stands for a reaction increasing reactivity (i.e., contributing to fuel consumption) and vice versa.

To investigate possible reasons for model shortcomings in predicting the effect of the equivalence ratio, we performed a sensitivity analysis of rate constants to fuel consumption at *T* = 950 K for the three mixtures experimentally investigated. As expected, results provided in Figure 11 do not highlight any possible modification capable of decreasing the reactivity of the rich mixture (φ = 2.0) while simultaneously increasing that of the lean mixture (φ = 0.5). Sensitivity coefficients have been normalized over that of the most sensitive reaction, Ḣ + O_2_ ↔ Ö + ȮH.

The competition between the branching reaction Ḣ + O_2_ ↔ Ö + ȮH and the third-body recombination Ḣ + O_2_ (+M) ↔ HȮ_2_ (+M) decreasing system reactivity increases for leaner mixtures. The first fuel-specific reaction appearing within the most sensitive reactions is the H-abstraction by O_2_ on pyrrole (C_4_H_5_N + O_2_ ↔ PYRLYL + HȮ_2_, reaction R19). As already highlighted in the above discussion, under these conditions, the reaction proceeds backward, consuming pyrrolyl and HȮ_2_ and, thus, decreasing fuel consumption. This negative effect is explained by the fact that, in addition to partly restoring the concentration of fuel, produced O_2_ is then converted through the recombination reaction Ḣ + O_2_ (+M) ↔ HȮ_2_ (+M) or O_2_ + HĊO ↔ HȮ_2_ + CO, forming once again HȮ_2_. Reactions belonging to the pyrrole subset, such as C_4_H_5_N + HȮ_2_ ↔ PYRLYL + H_2_O_2_ (reaction R18) and aĊ_3_H_4_CN + HȮ_2_ ↔ ȮH + Ċ_4_H_4_NO (reaction R48), convert this HȮ_2_ into more reactive radicals, thus increasing the reactivity. Another key reaction consuming HȮ_2_ is NO + HȮ_2_ ↔ ȮH + NO_2_, for which we adopted the value of Howard et al.,^71^ as already discussed by Song et al.^27^ As expected, H-abstraction reactions by ȮH and Ö also have a positive impact on reactivity. The isomerization of the resonance-stabilized pyrrolyl radical to the vinylic radical *c*Ċ_3_H_4_CN (reaction R43) clearly favors pyrrole conversion. The important role of Ċ_4_H_4_N isomer chemistry and, in particular, that of *c*Ċ_3_H_4_CN and aĊ_3_H_4_CN emerges clearly from the competition between the isomerization (aĊ_3_H_4_CN ↔ *c*Ċ_3_H_4_CN, reaction R42 backward) and oxidation (O_2_ + *c*Ċ_3_H_4_CN ↔ Ö + Ċ_4_H_4_NO, reaction R49) reactions increasing reactivity, with the decomposition reaction (*c*Ċ_3_H_4_CN ↔ C_2_H_2_ + ĊH_2_CN, reaction R46) consuming *c*Ċ_3_H_4_CN. The oxidation of aĊ_3_H_4_CN (aĊ_3_H_4_CN + HȮ_2_ ↔ ȮH + Ċ_4_H_4_NO, reaction R48) also contributes to increase the overall reactivity, forming two reactive radicals from relatively stable radicals.

**Figure 12:**
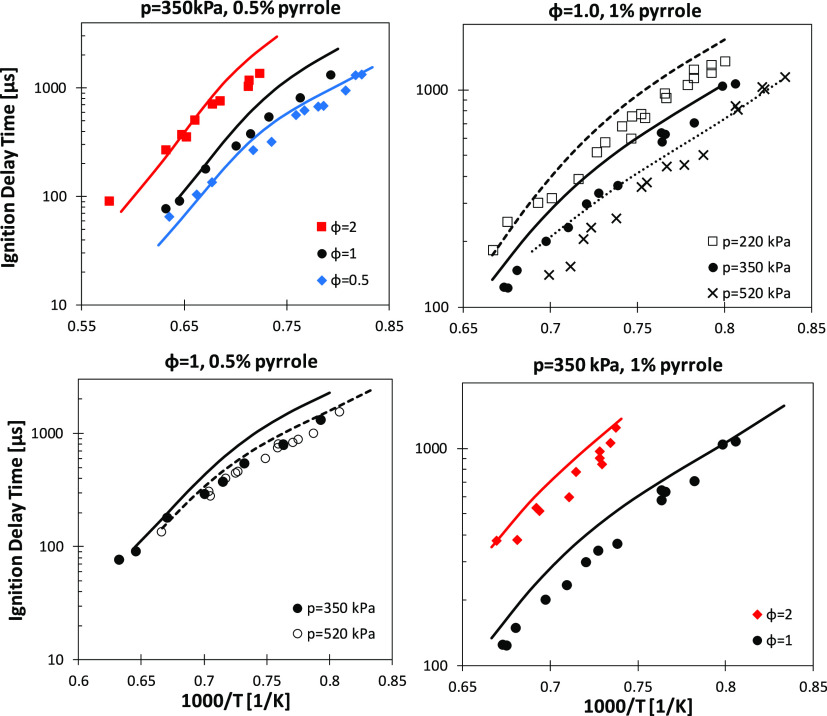
Shock-tube experimental (symbols)^35^ and simulated (lines) ignition delay times for highly diluted mixtures (>92.7 mol %) of pyrrole (0.5 and 1%) in O_2_ and argon at high temperatures.

MacNamara and Simmie^35^ studied pyrrole autoignition in a low-pressure shock tube. Figure 12 compares ignition delay time measurements for different pyrrole/oxygen/argon mixtures with model predictions.

**Figure 13:**
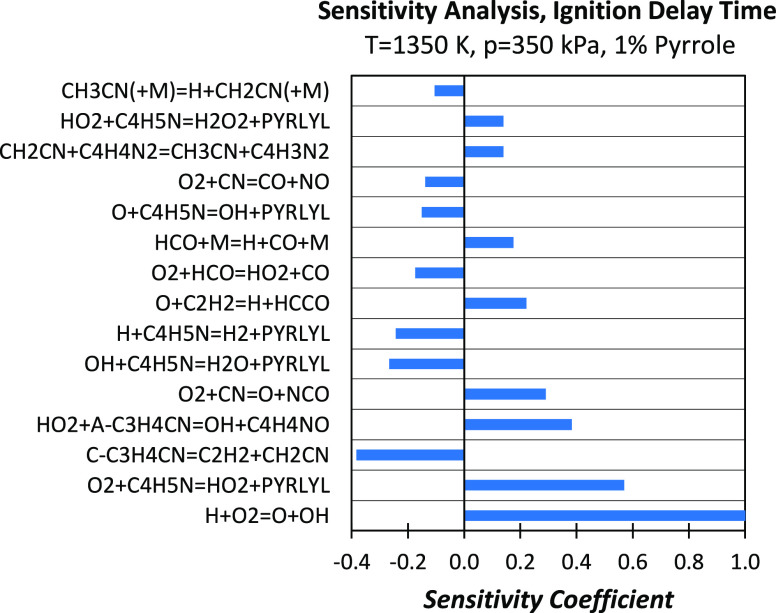
Sensitivity analysis of ignition delay times to rate constants for a stoichiometric 1% pyrrole/oxygen/argon mixture at *T* = 1350 K and *p* = 350 kPa. A positive sensitivity coefficient stands for a reaction promoting ignition and vice versa. Sensitivity coefficients have been normalized over that of the dominating reaction Ḣ + O_2_ ↔ Ö + ȮH.

Overall, simulated ignition delay times capture the effect of the pressure, equivalence ratio, and fuel concentration on the ignition propensity of pyrrole. Maximum deviations are as large as a factor of 1.7 in the worst cases. A sensitivity analysis has been carried out for the case of 1% pyrrole, *p* = 350 kPa, φ = 1, and *T* = 1350 K (Figure 13).

**Figure 14:**
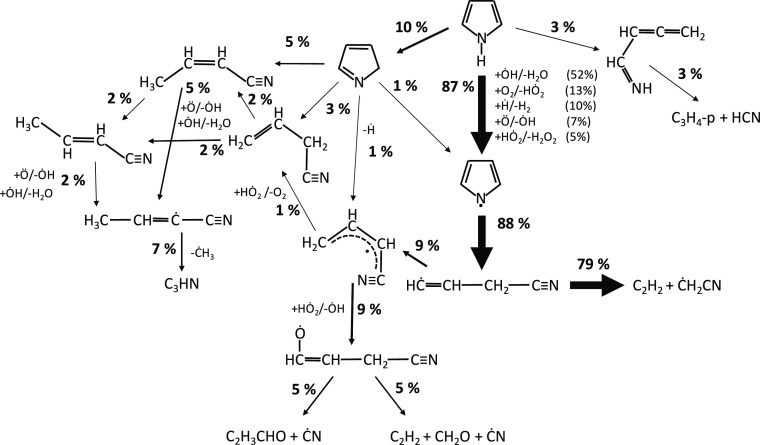
Rate of production analysis for a 1% pyrrole/O_2_/argon mixture at *T* = 1350 K, φ = 1.0, *p* = 350 kPa, and 20% fuel conversion (τ = 3.2 × 10^–4^ s). Arrow width qualitatively represent the importance of each reactive flux. Pathways with a flux going from or to an intermediate of <1% have been disregarded for clarity.

The H-abstraction reaction C_4_H_5_N + O_2_ ↔ PYRLYL + HȮ_2_ at this temperature condition proceeds in the forward direction, thus promoting fuel consumption and thereof ignition. Indeed, HȮ_2_ undergoes self-recombination to form H_2_O_2_ that is rapidly decomposed to form two hydroxyl radicals [HȮ_2_ + HȮ_2_ (+M) ↔ H_2_O_2_ + O_2_ (+M) and H_2_O_2_ (+M) ↔ 2ȮH (+M)] or recombines with Ḣ to directly produce two ȮH radicals (Ḣ + HȮ_2_ ↔ 2ȮH), promoting ignition. One of the major sources of Ḣ atoms is, together with formyl radical decomposition, the addition/elimination reaction involving cyanoacetylene (Ḣ + C_3_HN ↔ C_2_H_2_ + ĊN, reaction R47 backward).^72^ Other H-abstraction reactions (Ḣ + C_4_H_5_N, ȮH + C_4_H_5_N, and Ö + C_4_H_5_N) producing the pyrrolyl radical show a negative sensitivity coefficient, despite consuming the fuel. This is justified by the fact that pyrrolyl almost entirely isomerizes to *c*Ċ_3_H_4_CN that, at such high-temperature conditions, decomposes to acetylene and ĊH_2_CN that is resonance-stabilized and acts as a sink of Ḣ atoms, strongly inhibiting the occurrence of the branching reaction Ḣ + O_2_ ↔ Ö + ȮH that dominates high-temperature ignition. As shown in Figure 14, only a minor amount of *c*Ċ_3_H_4_CN isomerizes to aĊ_3_H_4_CN, whose oxidation (aĊ_3_H_4_CN + HȮ_2_ ↔ ȮH + Ċ_4_H_4_NO) promotes ignition.

**Figure 15:**
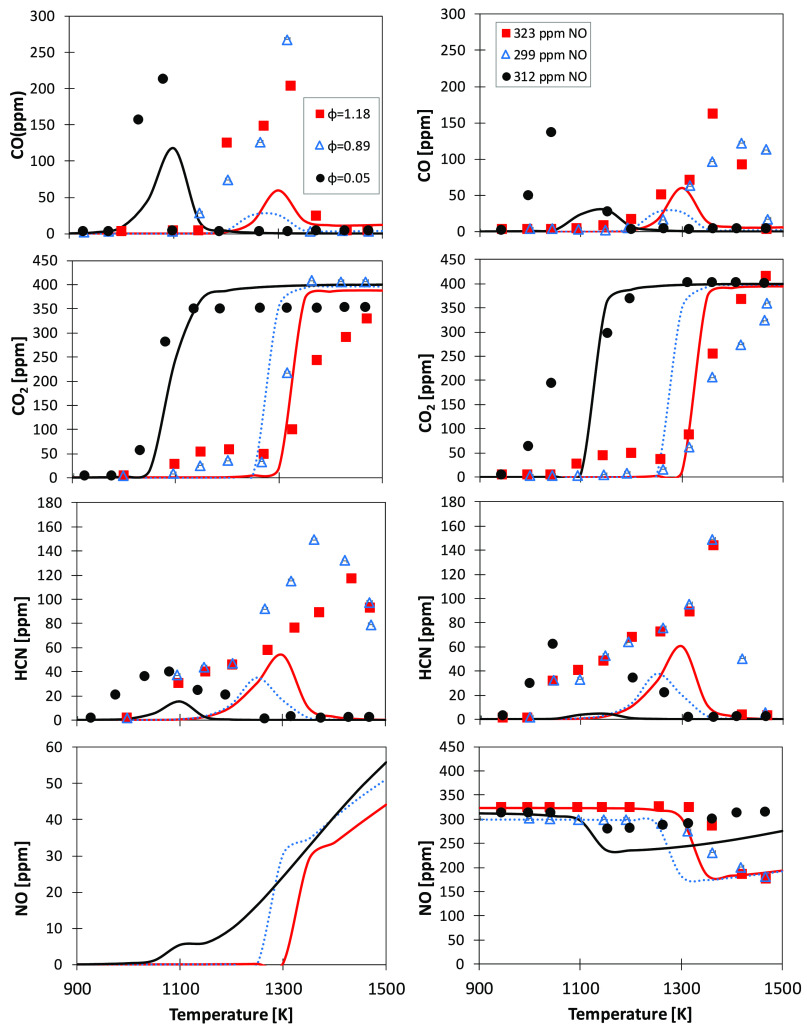
Speciation profiles from pyrrole (100 ppm) oxidation as a function of the temperature for different air excess ratios, without (left column) and with (right column) ∼300 ppm of NO addition. Residence time τ = 210/*T* s. Comparison between experimental data (symbols)^36^ and model predictions (lines).

To assess the governing chemistry of NO_*x*_ formation from fuel-bound nitrogen, Lumbreras et al.^36^ investigated the flow reactor oxidation of pyrrole in experiments with and without NO addition. Figure 15 compares model results to experimental data for the cases without NO (left column) and with NO (right column). The model qualitatively captures both the effect of the equivalence ratio on pyrrole oxidation with and without NO addition. However, except for the CO_2_ profiles, for which the reactivity is well-captured, large quantitative deviations (factor of ∼3 in the worst cases) exist for the peak concentrations of measured species, such as CO and HCN. For example, the model fails to predict the early formation of HCN in the leanest case (φ = 0.05), both with and without NO. Moreover, predicted CO formation is delayed, while NO consumption is anticipated. Despite a 5% uncertainty in the measurements declared by the authors, atomic balances highlight that some species that may be formed in significant quantities were not measured (e.g., C_2_H_2_, HNCO, C_2_H_4_, C_3_HN, and CH_3_CN), thus preventing any quantitative statement on model performances. Moreover, very little impact of model parameters was found when attempting to improve model performances. However, these data, together with those by Yamamoto et al.^39^ (Figure 17), are valuable to further assess the importance of formation and consumption pathways of HCN and NO, as discussed in the following. Despite not being measured, in Figure 15, we also report the temperature dependences of the NO mole fraction. As expected, the leanest and most reactive mixture shows the earliest and highest production of NO. The change of slope on the higher temperature end is due to the conversion of NO to NO_2_ through NO + Ö (+M) ↔ NO_2_ (+M) (reaction R56), facilitated by the branching reaction Ḣ + O_2_ ↔ Ö + ȮH providing high amounts of oxygen atoms.

**Figure 16:**
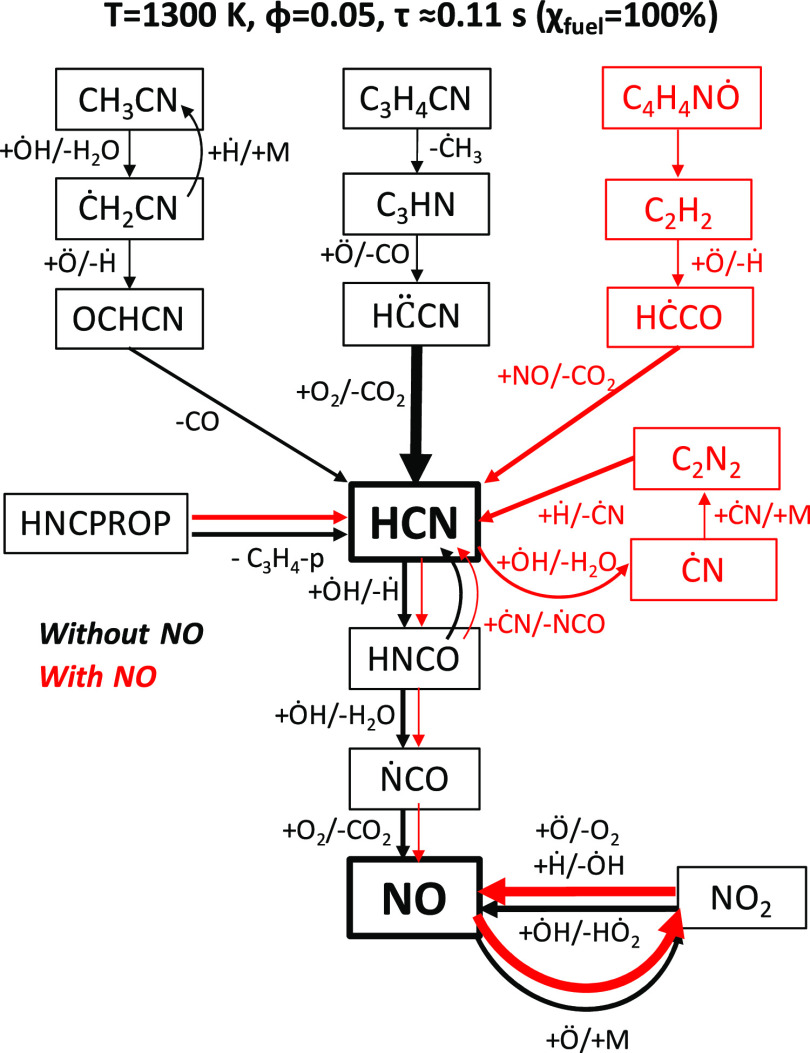
Main HCN and NO formation pathways for φ = 0.05 mixtures of Figure 15 with (red) and without (black) NO addition.

Figure 16 shows the main formation and consumption pathways forming HCN and NO, also focusing on the conversion of major nitrogen-containing intermediates, such as HCN, CH_3_CN, and Ċ_3_H_4_CN, to NO. In the case without NO addition (left column of Figure 15), HCN is mostly formed by the successive decomposition steps of Ċ_3_H_4_CN, undergoing β-scission to cyanoacetylene (C_3_HN) that is converted to the cyanomethylene radical (H*C̈*CN) through reaction R57 and then to HCN through reaction R58. A secondary pathway, of similar importance in the case of NO addition, is the direct decomposition of allenic imine HNCPROP to HCN and propyne. The channel of lower importance involves acetonitrile (CH_3_CN), forming the cyanomethyl radical (ĊH_2_CN) through reaction R59. ĊH_2_CN is subsequently converted to formyl cyanide (OCHCN) via reaction R60. Formyl cyanide then eliminates CO to yield HCN (reaction R61). ȮH is added to HCN and eliminates Ḣ to form isocyanic acid (HNCO, reaction R62) that through H-abstraction by ȮH in reaction R64 forms the isocyanato radical (ṄCO). ṄCO reacts with O_2_, which is present in large excess, to form NO and CO_2_ (reaction R65). NO is further converted to NO_2_ through the third-body reaction NO + Ö (+M) ↔ NO_2_ (+M) and then regenerated from NO_2_ by means of addition/elimination reactions.

In the case of NO addition (right column of Figure 15), different pathways lead to the formation of HCN. C_4_H_4_NȮ decomposition to acetylene according to the lumped reaction R51 (C_4_H_4_NȮ → C_2_H_2_ + CH_2_O + ĊN) yields the ethynyloxy radical (HĊCO) through C_2_H_2_ + Ö ↔ Ḣ + HĊCO. HĊCO is transformed to HCN by means of reaction R68: HĊCO + NO ↔ HCN + CO_2_. HCN then undergoes H-abstraction by ȮH or ȮH addition (reactions R62 and R63). The first channel leads to the formation of the cyano radical (ĊN) that after self-recombination (reaction R66) produces cyanogen (C_2_N_2_). Cyanogen is converted back to HCN by Ḣ addition in reaction R67, producing once again the cyano radical. The H-abstraction pathway leads to the same formation route of NO previously discussed for the case without NO. The presence of NO in the feed triggers NO conversion to NO_2_ that is once again converted back to produce NO. From this reaction cycle, it is possible to explain the successive consumption and formation of NO observed in the right column of Figure 15.

**Figure 17:**
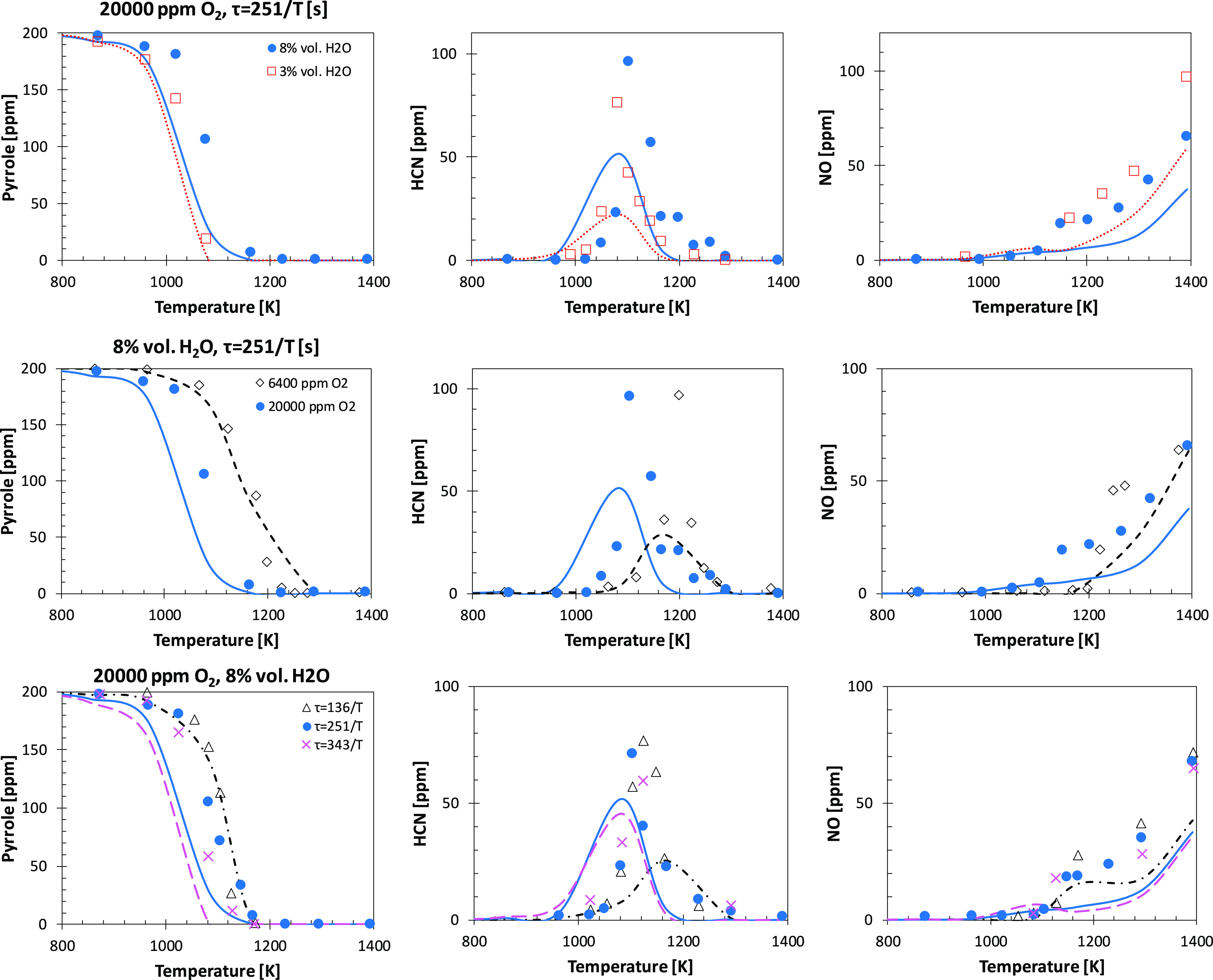
Comparison of experimental data to model predictions for pyrrole, HCN, and NO under different conditions of H_2_O concentration (top row), O_2_ concentration (central row), and residence time (bottom row). Symbols are experimental data,^39^ and lines are model predictions.

Figure 17 compares model predictions with the flow reactor measurements by Yamamoto et al.^39^ The largest deviations are observed for the lean cases (20 000 ppm of O_2_, i.e., φ = 0.06 without considering the H_2_O content) when 8% H_2_O is added to the system. Indeed, the model overestimates the reactivity by ∼50 K. The conditions of these experiments are comparable to those of Lumbreras et al.,^36^ where the model showed a similar deviation in terms of the temperature but in the opposite direction. Therefore, we consider model performances to be in reasonable agreement with the experimental data because no modification to the kinetics would lead to improvement for both sets of data. It should also be noted that H_2_O does not play any role in terms of modifications to the radical pool, as evident from the inhibiting effect on reactivity rather than an increase that would be expected from, for example, higher yields of ȮH. However, the collisional efficiency of water (i.e., ∼6–12 times higher than that of N_2_^55^) strongly promotes the chain propagation reaction [Ḣ + O_2_ (+M) ↔ HȮ_2_ (+M)] and termination reaction [HȮ_2_ + HȮ_2_ (+M) ↔ H_2_O_2_ + O_2_ (+M)] over chain branching (i.e., Ḣ + O_2_ ↔ Ö + ȮH) in the H_2_/O_2_ subset in the temperature window where the onset of fuel conversion is observed (*T* = 1000–1100 K), thus reducing the overall reactivity. Model performances would benefit from a better assessment of pressure dependence of primary fuel reactions largely discussed in section 6.1 and from a more rigorous implementation of collisional efficiencies in the current formalism for pressure-dependent rate expressions. Model results agree better for the remaining cases, i.e., 20 000 ppm of O_2_ with 3% H_2_O addition and 6400 ppm of O_2_ (φ = 0.2) with 8% H_2_O addition. Notably, the model correctly predicts fuel conversion for short residence times (τ = 136/*T* s), even for the leanest case with 8% H_2_O addition. In general, the model captures very well the dependence of HCN and NO formation trends and the relative magnitude of their peaks on operating conditions.

## 7 Conclusion

In this work, the pyrolysis and oxidation of pyrrole were experimentally investigated in an atmospheric pressure JSR, significantly extending the validation targets available for pyrrole kinetic model validation purposes. A preliminary model based on previous research efforts and analogy with kinetic subsets already implemented in the CRECK kinetic framework is presented, showing generally good agreement after some adjustment on available kinetic parameters, within their uncertainties. To the knowledge of the authors, this pyrrole model is the first model comprehensively validated in the literature and allows for the inclusion of pyrrole as a representative nitrogen-containing component in more complex surrogate models of pyrolysis bio-oils.^11^ Moving from a detailed kinetic analysis aimed at highlighting reasons for model deviations and existing shortcomings, we believe that theoretical, experimental, and kinetic modeling efforts should be devoted to the following: (1) There should be better assessment of temperature- and pressure-dependent kinetics of isomerization channels to pyrrolenine, crotonitrile isomers, and allyl cyanide that initiate and dominate pyrrole decomposition kinetics. Available theoretical information on the potential energy surfaces are indeed quite accurate and detailed to be reproduced with current state-of-the-art electronic structure methods and multi-well master equation solvers. (2) H-abstraction reactions consuming pyrrole, pyrrolenine, and their C_3_H_5_CN isomers are currently largely based on analogy rules with systems, leading to resonance-stabilized radicals (e.g., allylic type radical) not containing nitrogen, whose influence is expected to be significant. Such rate coefficients should be theoretically re-evaluated with more accuracy. (3) Secondary reactivity of derived radicals of such an unsaturated system can be complicated by resonance stabilization. For example, recombination reactions of the pyrrolyl radical and HO_2_ have been included in our kinetic model, largely on the basis of analogy with cyclopentadienyl chemistry and/or allyl radical chemistry. However, no impact of such pathways was observed at the conditions where experimental data are available at present. Clearly, this observation poses some question on the general validity of the analogy rules adopted in this work and in previous kinetic modeling studies. Similar observations apply to the interactions between the cyano allyl radical (aĊ_3_H_4_CN) and its vinyl isomers (*c*Ċ_3_H_4_CN and Ċ_3_H_4_CN) with O_2_ and HO_2_, where further theoretical investigations would be beneficial to increase model predictive capabilities. (4) The deficit in N atoms highlighted by the atomic balances should be investigated thoroughly. A valuable perspective would be to perform such a study with an advanced diagnostic tool, like time-of-flight mass spectrometry with more direct sampling using, for example, a molecular beam to minimize the loss of species during the sampling. This technique would also allow for the detection of species having relatively low stabilities, like pyrrolyl and other resonance-stabilized radicals, which play a central role in the gas-phase chemistry of pyrrole. (5) Experimental data on pyrrole combustion at a higher pressure would be useful to extend the confidence of the proposed model at conditions closer to that of real combustion devices (e.g., turbines). In particular, ignition delay time data of fuel/air mixtures in a high-pressure shock tube would be useful, although the low volatility of pyrrole might inhibit tests in non-diluted mixtures. In addition, flame data are only available at a very low pressure (i.e., 0.032 atm^38^), where pressure-dependent kinetics are extremely important. Indeed, such targets have not been reported in the validation of the present kinetic model, where we only adopt high-pressure limit rate constants. However, the understanding of N fuel chemistry would benefit from laminar flame speed measurements at atmospheric pressure. (6) On the basis of past tests of bio-oil use at the industrial scale,^6^ where pilot flames fed with conventional hydrocarbons are often used, it would be interesting to assess the kinetic effects of hydrocarbon fuels doped with pyrrole. It is important to note that, for this type of test, the possible importance of cross chemical interactions of resonance-stabilized radicals from pyrrole decomposition and oxidation chemistry with other components in the mixtures should be assessed. In addition to the impact on macroscopic reactivity targets (e.g., ignition delay times and laminar flame speed), speciation measurements on NO_*x*_ formation and polycyclic aromatic hydrocarbon (PAH) growth should be performed. Indeed, as highlighted in this study, acetylene and other unsaturated hydrocarbons are produced in large quantities and may significantly contribute to molecular growth kinetics, posing some question on the pollution potential of bio-oils not only in terms of nitrogen oxides but also for PAH and particulate matter formation.

## Supporting Information Available

Experimental data from JSR measurements (XLSX)
Thermodynamic properties of relevant species, SMILES identifiers, and structural data from theoretical calculations (PDF)
Model 1 kinetics: pyrrole pyrolysis and oxidation (189 species, 2888 reactions, core chemistry + NO_*x*_ subset + pyrrole subset) (TXT)
Model 1 thermo: pyrrole pyrolysis and oxidation (189 species, 2888 reactions, core chemistry + NO_*x*_ subset + pyrrole subset) (TXT)
Model 2 kinetics: high-temperature CRECK model with NO_*x*_ and pyrrole chemistry (500 species and 17 472 reactions) containing the kinetic subset of reference species of possible interest for bio-oil surrogates (e.g., aldehydes, alcohols, organic acids, phenolic species and oxygenated aromatics with multiple substitutions, and methyl esters) (TXT)
Model 2 thermo: high-temperature CRECK model with NO_*x*_ and pyrrole chemistry (500 species and 17 472 reactions) containing the kinetic subset of reference species of possible interest for bio-oil surrogates (e.g., aldehydes, alcohols, organic acids, phenolic species and oxygenated aromatics with multiple substitutions, and methyl esters) (TXT)
Pyrrole pyrolysis and oxidation subset with detailed references on selected rate coefficients (TXT)
