# Supplementary material for: Pyrolysis and Combustion Chemistry of Pyrrole, a Reference Component for Bio-oil Surrogates: Jet-Stirred Reactor Experiments and Kinetic Modeling
Source: Energy Fuels. 2021 Mar 2;35(9):7265–84. doi: 10.1021/acs.energyfuels.0c03874 (PMC8161689; doi:10.1021/acs.energyfuels.0c03874)
Supplement: Supplementary file 2 — ef0c03874_si_002.pdf [file ef0c03874_si_002.pdf]

Supporting Information

Pyrolysis and combustion chemistry of pyrrole, a reference component for bio-oil surrogates. Jet-stirred reactor experiments and kinetic modelling.

[1,*]Matteo Pelucchi, [2]Suphaporn Arunthanayothin, [2,3]Yu Song, [2,*]Olivier Herbinet, [1]Alessandro Stagni,

[4, 5]Hans-Heinrich Carstensen, [1]Tiziano Faravelli, [2]Frédérique Battin-Leclerc

[1] CRECK Modeling Lab, Department of Chemistry Materials and Chemical Engineering, Politecnico di Milano, Italy
[2] Laboratoire Réactions et Génie des Procédés, CNRS, Université de Lorraine, ENSIC, Nancy Cedex, France
[3]University Orléans, INSA-CVL, PRISME, EA 4229, F45072 Orléans, France
[4] Fundación Agencia Aragonesa para la Investigación y Desarrollo (ARAID), Zaragoza, Spain
[5] Department of Chemical and Environmental Engineering, Engineering and Architecture School, University of Saragoza, Spain

Corresponding authors:

Model:
Dr. Matteo Pelucchi,
Email: matteo.pelucchi@polimi.it

Experiments:
Dr. Olivier Herbinet,
Email: olivier.herbinet@univ-lorraine.fr

Thermodynamic Properties

Table S1 reports thermodynamic properties for the species listed in Table 1 of the main text. The names given are those used in the kinetic model. Again, refer to Table 1 of the manuscript for the regular names. The data for pyrrole, pyrrolenine and pyrrolyl radical have been calculated in this work at the CBS-QB3 level of theory. Enthalpies in parenthesis are from the ATcT version 1.122o database [1]. These data are generally close to the enthalpies stored in the NASA polynomials, proving the good quality of the thermodynamic database used in this work for these species. Adopting the same methods discussed in the manuscript, we also computed thermodynamic parameters for NCCO, OCHCN and OCH_2CN.

Table S1: Thermodynamic properties for the species listed in Table 1 of the main text. Species whose thermodynamic properties have been computed in this work are highlighted in blue.

	**ΔH_f (T=298 K)**	**S (T=298 K)**	**Cp (T[K])** [cal/mol/K]						
**Species**	*[kcal/mol]*	*[cal/mol·K]*	*300*	*400*	*500*	*600*	*800*	*1000*	*1500*
Pyrrole	25.3	64.6	17.1	23.1	27.9	31.8	37.5	41.4	47.2
Pyrrolenine	40.6	66.1	16.5	22.5	27.4	31.4	37.4	41.5	47.5
C-C_3H_5CN	32.0	71.6	19.3	24.0	28.0	31.5	36.8	40.7	46.7
T-C_3H_5CN	33.6	71.6	19.6	24.3	28.3	31.7	37.0	40.8	46.8
A-C_3H_5CN	38.0	74.4	22.5	27.1	31.0	34.2	39.1	42.8	49.1
HNCPROP	68.7	74.6	20.5	25.6	29.8	33.3	38.5	42.0	46.0
Pyrrolyl	69.3	65.7	16.5	22.0	26.4	29.9	34.8	38.1	43.0
C_3H_4CN	84.9	72.1	21.7	25.9	29.4	32.1	36.1	38.5	41.1
C-C_3H_4CN	91.5	80.1	21.8	25.7	28.9	31.5	35.4	37.9	40.8
A-C_3H_4CN	68.2	73.6	15.6	21.9	26.9	30.9	36.3	39.5	43.2
C_4H_4NO	74.4	80.6	25.1	29.9	33.8	37.0	41.8	45.0	49.1
CH_3CN	17.7 (17.7)	58.1	12.6	14.7	16.6	18.3	21.3	23.5	27.0
CH_2CN	62.9 (62.3)	59.9	13.1	14.7	16.1	17.3	19.2	20.6	22.8
CH_2CHCN	44.0 (44.7)	62.9	14.4	17.2	19.6	21.7	25.0	27.5	31.2
CHCHCN	105.8 (106.5)	65.0	14.3	16.6	18.6	20.2	22.6	24.2	26.9
C_3HN	88.0	59.3	15.0	17.0	18.4	19.4	20.9	22.1	24.0
$C_4H_4N_2$	50.1	77.7	22.3	27.0	30.9	34.1	39.0	42.3	47.3
$C_4H_3N_2$	91.2	80.5	24.6	27.2	30.0	32.7	37.4	40.7	44.3
$C_4H_2N_2$	80.9	71.6	21.0	24.8	28.0	30.5	34.3	36.7	39.0
C_2H_5CN	12.7	68.1	17.4	21.1	24.3	27.1	31.6	35.1	40.7
CH_2CH_2CN	58.2	68.5	13.4	17.9	21.7	24.9	29.9	33.3	37.3
CH_3CHCN	54.2	69.5	16.6	18.7	21.1	23.6	28.4	32.2	37.0
CH_2N	57.0 (57.0)	53.6	9.1	10.2	11.2	12.2	13.8	15.1	17.1
HCCN	113.9 (115.5)	59.2	12.3	13.4	14.3	14.9	16.0	16.8	18.0
C_2N	*173.4*	57.1	9.5	10.3	11.0	11.6	12.4	12.8	13.4

	(164.9)								
C_4N_2	126.5	69.4	20.6	22.8	24.3	25.4	27.1	28.4	30.5
NCCO	50.4	63.2	11.5	12.4	13.1	13.7	14.8	15.6	16.6
OCHCN	11.5	64.8	13.4	14.9	16.3	17.8	19.7	21.1	23.2
OCH_2CN	43.9	67.2	14.9	16.9	19.0	21.0	23.6	25.5	28.2
CN	104.8 (105.2)	48.4	7.0	7.0	7.2	7.3	7.7	8.0	8.5
HCN	*31.0 (30.9)*	48.2	8.6	9.4	10.0	10.5	11.3	12.0	13.2
HNCO	-28.3 (-28.5)	56.9	10.8	12.1	13.2	14.0	15.2	16.1	17.6
NCO	30.6 (30.5)	55.5	9.6	10.4	11.2	11.8	12.7	13.3	14.1

Species nomenclature and chemical identifiers (SMILES)

Table S2 reports SMILES identifiers for the chemical species described in the kinetic model. Structures, model name and molecular formula are also reported in Table 1 of the manuscript.

Table S2: SMILES identifiers for species in the kinetic model (Table 1 main text).

Species Name	Model name	SMILES
Pyrrole	C_4H_5N	C1=CNC=C1
Pyrrolenine	PYRLNE	C1CNC=C1
Cis-crotonitrile	C-C_3H_5CN	CC=CC#N
Trans-crotonitrile	T-C_3H_5CN	CC=CC#N
Allyl cyanide	A-C_3H_5CN	C=CCC#N
Allenic Imine	HNCPROP	C=C=CC=N
Pyrrolyl radical	PYRLYL	C1=C[CH]N=C1
Cyano propen-2yl radical	C_3H_4CN	CC=[C]C#N
Cyano propen-4yl radical	C-C_3H_4CN	[CH]=CCC#N
Cyano allyl radical	A-C_3H_4CN	[CH]=CCC#N
Allyloxy cyanide	C_4H_4NO	[CH2]C=CC#N
Acetonitrile	CH_3CN	CC#N
Cyano methyl radical	CH_2CN	[CH2]C#N
Acrilonitrile	CH_2CHCN	C=CC#N
Acrilonitrile radical	CHCHCN	[CH]=CC#N
Cyanoacetylene	C_3HN	C#CC#N
Butanedinitrile	$C_4H_4N_2$	C(CC#N)C#N
Butanedinitrile radical	$C_4H_3N_2$	[CH](CC#N)C#N
Fumaronitrile	$C_4H_2N_2$	C(=CC#N)C#N
Propionitrile	C_2H_5CN	CCC#N
Propionitrile primary radical	CH_2CH_2CN	[CH2]CC#N
Propionitrile secondary radical	CH_3CHCN	C[CH]C#N
Formimidoyl	CH_2N	[CH]=N
Cyanomethylene radical	HCCN	[CH]=C=[N]
Cyanomethylidyne	C_2N	[C]#C[N]
2-Butynedinitrile	C_4N_2	C(#CC#N)C#N
Carbonyl cyanide	NCCO	O=C=C=[N]

Formyl cyanide	OCHCN	C(=O)C#N
Hydroxy acetonitrile radical	OCH_2CN	C(#N)C[O]
Cyano radical	CN	[C]#N
Hydrogen cyanide	HCN	C#N
Isocyanic acid	HNCO	C(=N)=O
Isocyanate radical	NCO	C(#N)[O]

Electronic structure calculation outputs for major species in pyrrole pyrolysis and oxidation

Table S3 reports enthalpies of formation, geometries, rotational constants and frequencies for pyrrole, pyrrolenine and pyrrolyl radical. Pyrrolenine is a lumped species representing two molecules and pyrrolyl represents three radicals. Although the results for all these species are given, those of lowest energy (bold faced) are used as representative species in the model.

Table S3: enthalpies of formation, geometries, rotational constants and frequencies for pyrrole, pyrrolenine and pyrrolyl radical.

**Name**	**Structure**<br><br>**Enthalpy of formation [kJ/mol]**	**geometry**	**Frequencies [cm-1]**<br><br>***Rotational constants***
Pyrrole	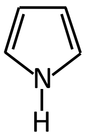<br><br>**106.1**	6 0.031183 -0.057752 0.019942<br>6 0.037879 -0.003414 1.395032<br>6 1.398236 -0.003415 1.815282<br>6 2.179350 -0.057754 0.683566<br>7 1.338194 -0.090452 -0.402230<br>1 1.634766 -0.132005 -1.362236<br>1 -0.784594 -0.075646 -0.684229<br>1 -0.834591 0.032308 2.028554<br>1 1.761372 0.032306 2.830510<br>1 3.250154 -0.075642 0.562212	471.497 630.818 640.569<br>686.205 727.553 830.459<br>878.300 881.402 902.207<br>1034.108 1066.866 1092.450<br>1158.876 1174.042 1308.958<br>1417.799 1455.734 1500.529<br>1577.040 3228.792 3239.955<br>3255.653 3261.739 3674.884<br><br>*Rotational Constants [GHz] = 4.546 9.031 9.155*
Pyrrolenine	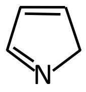<br><br>**170.0**	6 0.102783 0.074261 -0.115063<br>6 0.052080 0.037529 1.383932<br>6 1.321179 -0.021248 1.821104<br>6 2.170257 -0.022102 0.621175<br>7 1.516736 0.030890 -0.486119<br>1 -0.419770 -0.772371 -0.577968<br>1 -0.850981 0.056003 1.978662<br>1 1.669602 -0.060689 2.844178<br>1 3.256277 -0.062986 0.629270<br>1 -0.354210 0.980211 -0.532843	365.882 553.682 705.521<br>838.135 860.143 909.145<br>931.194 946.280 975.493<br>993.500 1000.159 1117.414<br>1161.221 1261.179 1310.380<br>1370.555 1410.901 1540.989<br>1648.427 3023.996 3047.449<br>3153.541 3204.193 3226.673<br><br>*Rotational Constants [GHz] = 4.496 8.417 9.111*
	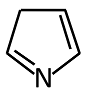<br><br>175.1	6 0.070683 0.006269 -0.028759<br>6 -0.023094 -0.032488 1.472994<br>6 1.433131 -0.029049 1.834944<br>6 2.122205 0.005998 0.683896<br>7 1.278934 0.027801 -0.467094<br>1 -0.780513 0.015926 -0.702458<br>1 -0.571765 0.831969 1.868963<br>1 1.828712 -0.050996 2.839573<br>1 3.193767 0.018857 0.541414<br>1 -0.556609 -0.925167 1.824572	349.961 541.565 713.283<br>777.602 843.910 855.348<br>937.769 941.693 963.492<br>967.474 1022.355 1123.004<br>1133.321 1263.562 1295.328<br>1351.658 1405.971 1565.436<br>1630.370 3019.602 3046.192<br>3167.297 3212.634 3238.036<br><br>*Rotational Constants [GHz] = 4.499 8.574 8.946*
Pyrrolyl	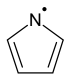	6 0.071921 -0.000004 -0.023469<br>6 0.014505 -0.000006 1.435564<br>6 1.311680 -0.000007 1.843518<br>6 2.099736 -0.000002 0.614268<br>7 1.332465 0.000000 -0.488771	491.813 543.803 655.880<br>714.423 822.984 839.479<br>882.915 912.888 934.245<br>1042.915 1075.698 1091.244<br>1204.322 1291.300 1355.879

	**290.2**	1 -0.772323 -0.000006 -0.702642	1434.007 1560.655 3192.606
		1 -0.879937 -0.000011 2.040368	3196.847 3225.590 3245.387
		1 1.698949 -0.000006 2.851406	
		1 3.180753 0.000003 0.540585	*Rotational Constants [GHz] = 4.682 8.806 9.999*
	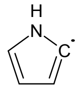	7 -0.010622 0.000005 -0.008119	297.562 455.964 620.349
		6 -0.004477 -0.000002 1.378298	684.433 750.635 855.024
		6 1.307783 -0.000009 1.787023	857.079 890.698 1023.335
		6 2.147772 -0.000006 0.622727	1059.497 1106.003 1172.759
		6 1.279552 0.000019 -0.431226	1253.052 1363.382 1407.718
		1 -0.924977 0.000009 1.938975	1457.069 1553.542 3226.346
		1 1.650194 -0.000013 2.810853	3255.339 3261.472 3676.725
	392.8	1 3.224247 -0.000017 0.590085	
		1 -0.825601 0.000028 -0.597207	*Rotational Constants [GHz] = 4.764 8.900 10.253*
	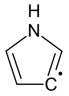	6 -0.013287 0.000004 -0.010705	436.274 539.736 637.077
		7 -0.001948 0.000012 1.372728	650.861 739.938 830.403
		6 1.291255 0.000005 1.833475	864.505 889.356 1035.865
		6 2.146385 -0.000008 0.746121	1065.748 1151.535 1174.812
		6 1.301651 -0.000008 -0.382283	1237.931 1377.965 1432.437
		1 1.507657 0.000009 2.890112	1475.700 1535.385 3238.018
		1 3.223553 -0.000016 0.780135	3256.583 3267.945 3657.914
	391.5	1 -0.930106 0.000008 -0.574662	
		1 -0.821213 0.000021 1.957725	*Rotational Constants [GHz] = 4.782 8.952 10.267*

References

[1] B. Ruscic and D. H. Bross, Active Thermochemical Tables (ATcT) values based on ver. 1.122o of the Thermochemical Network (2020); available at ATcT.anl.gov
